# Supplementary material for: 50 Years of Antibody Numbering Schemes: A Statistical and Structural Evaluation Reveals Key Differences and Limitations
Source: Antibodies (Basel). 2024 Dec 4;13(4):99. doi: 10.3390/antib13040099 (PMC11672675; doi:10.3390/antib13040099)
Supplement: Supplementary file 1 [file antibodies-13-00099-s001.zip › antibodies-3266626-supplementary.pdf]

SUPPLEMENTARY INFORMATION

50 years of antibody numbering schemes: a statistical and structural evaluation reveals key differences and limitations

Zirui Zhu[1,2], Katherine S. Olson[1], and Thomas J. Magliery[1,*]

[1]Department of Chemistry and Biochemistry, The Ohio State University, Columbus, Ohio, 43210, United States of America
[2]Chemistry Graduate Program, The Ohio State University

[*]Corresponding author: The Ohio State University, 151 W. Woodruff Ave., Columbus, OH 43210; email: magliery.1@osu.edu; phone: +1 (614) 247-8425

Items included in this document:

1) Supplementary Figure S1a: Conservation for selected residues in the variable light domain
2) Supplementary Figure S1b: Conservation for selected residues in the variable heavy domain
3) Supplementary Figure S2a: Distribution of LCDRs length for numbering schemes
4) Supplementary Figure S2b: Distribution of HCDRs length for numbering schemes
5) Supplementary Figure S3: Structural comparison of the lambda and kappa light chains
6) Supplementary Figure S4: Structural alignment of the CDR loops from multiple antibody variable domains
7) Supplementary Figure S5a: A pivot point in the LCDR1 is located at L30 in the lambda light chain
8) Supplementary Figure S5b: Another pivot point in the LCDR1 is located at L29 in the kappa light chain
9) Supplementary Figure S6: Amino acid distribution in each CDR loop compared to reference distribution in yeast
10) Supplementary Figure S7: The amino acid distribution is similar for both mouse and human antibodies
11) Supplementary Figure S8: Box-and-whisker plot of the regional relative entropy differences between mouse and human variable domains
12) Supplementary Figure S9: Amino acids distribution at pivot points in mouse and human antibody variable domains
13) Supplementary Figure S10: The residue-wise relative entropy and the AbNatiV Score calculated based on the consensus mouse sequence in the variable domain
14) Supplementary Figure S11a: A representation of the consensus sequence, consensus structure, and main-chain hydrogen bonding pattern of human immunoglobulin variable light domains
15) Supplementary Figure S11b: A representation of the consensus sequence, consensus structure, and main-chain hydrogen bonding pattern of human immunoglobulin variable heavy domains
16) Supplementary Table S1: Table of Antibody Variable Domains Amino Acids Distribution and the Codon Usage in Yeast
17) Supplementary Table S2: Table of Relative Entropy Calculated in each Region in the Variable Domains

18) Supplementary Table S3: Comparison of the CDR definition of different numbering schemes for immunoglobulin variable domains
19) Supplementary Table S4a: Table of Antibody Template PDB Entries, Organism, Light Chain Type and Reference Information
20) Supplementary Table S4b: Table of Antibody Template Protein Amino Acid Sequences
21) Supplementary Table S5a: Table of Antibody Template Protein LCDRs Amino Acid Sequences
22) Supplementary Table S5b: Table of Antibody Template Protein HCDRs Amino Acid Sequences
23) Supplementary Table S6: Table of Sequential and Structural Conserved Residues in CDRs
24) Supplementary Table S7: Table of Antibody Template Protein Light Chain Amino Acid Sequences
25) Supplementary Table S8a: Table of Relative Entropy of the Residues in LCDR1 based on different numbering schemes
26) Supplementary Table S8b: Table of Relative Entropy of the Residues in HCDR1 based on different numbering schemes
27) Supplementary Table S9a: Table of the Average Relative Entropy Calculated in each Regions in the Variable Domains between Human and Mouse
28) Supplementary Table S9b: Table of the Average, Median, Standard Deviation of the Relative Entropy Calculated and outliers in each Regions in the Variable Domains between Human and Mouse
29) Supplementary Table S10: Table of the Amino Acid Distributions at the Outlier Residues in Human and Mouse
30) Supplementary Table S11: Table of the Residue Relative Entropy, Consensus Mouse variable domain Sequences and Calculated AbNatiV Score based on the consensus sequence

Figure S1a

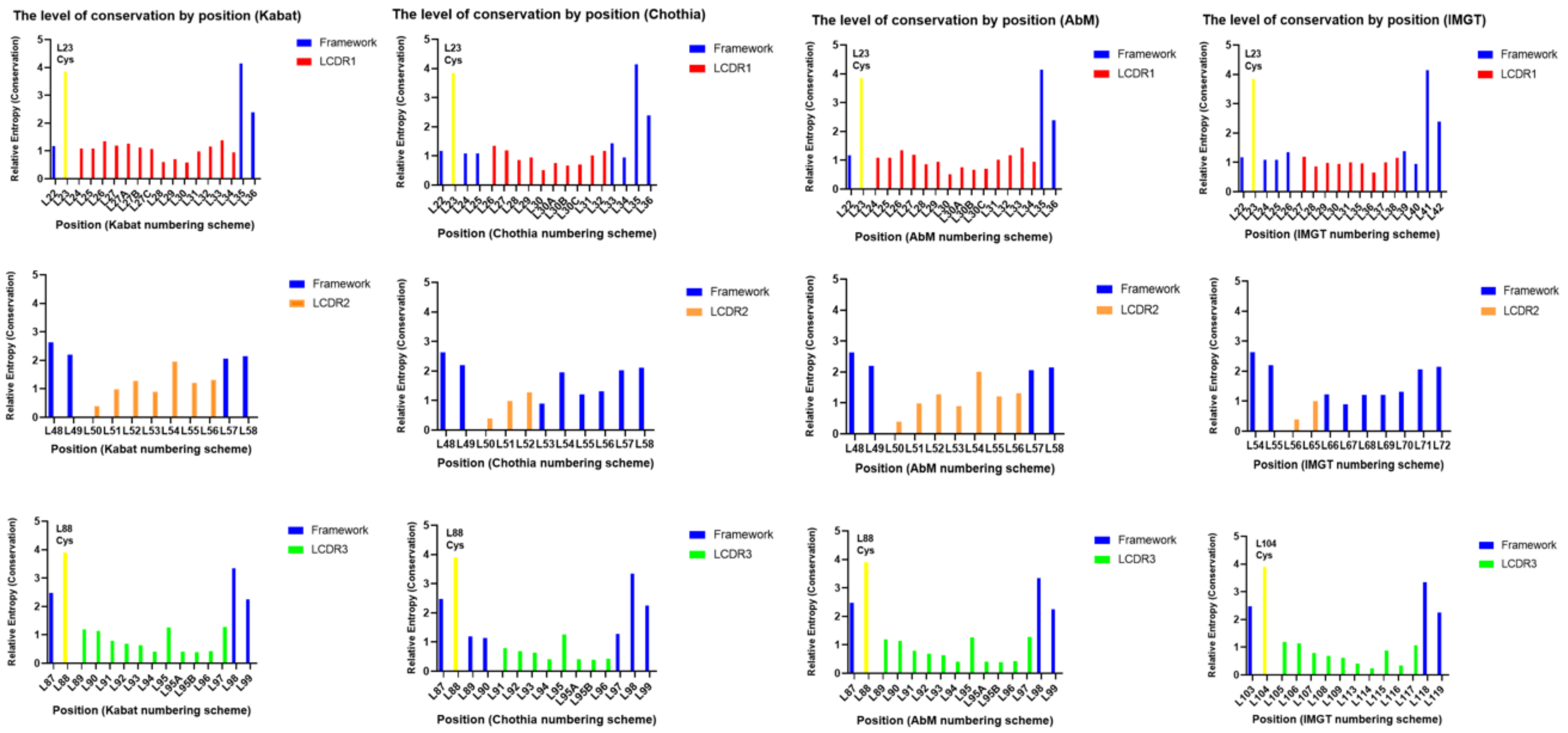

Supplemental Figure S1a. Conservation for selected residues in the variable light domain. The conservation was calculated for each residue as the relative entropy between the positional distribution and the all-position reference state, with residue position given by the Kabat, Chothia, AbM and IMGT numbering schemes. Relative entropies of residues within the CDR are colored according to the LCDR to which they belong (LCDR1 shown in red, LCDR2 in orange, and LCDR3 in green), while framework residues are shown in blue. Highlighted cysteine residues are shown in yellow.

Figure S1b

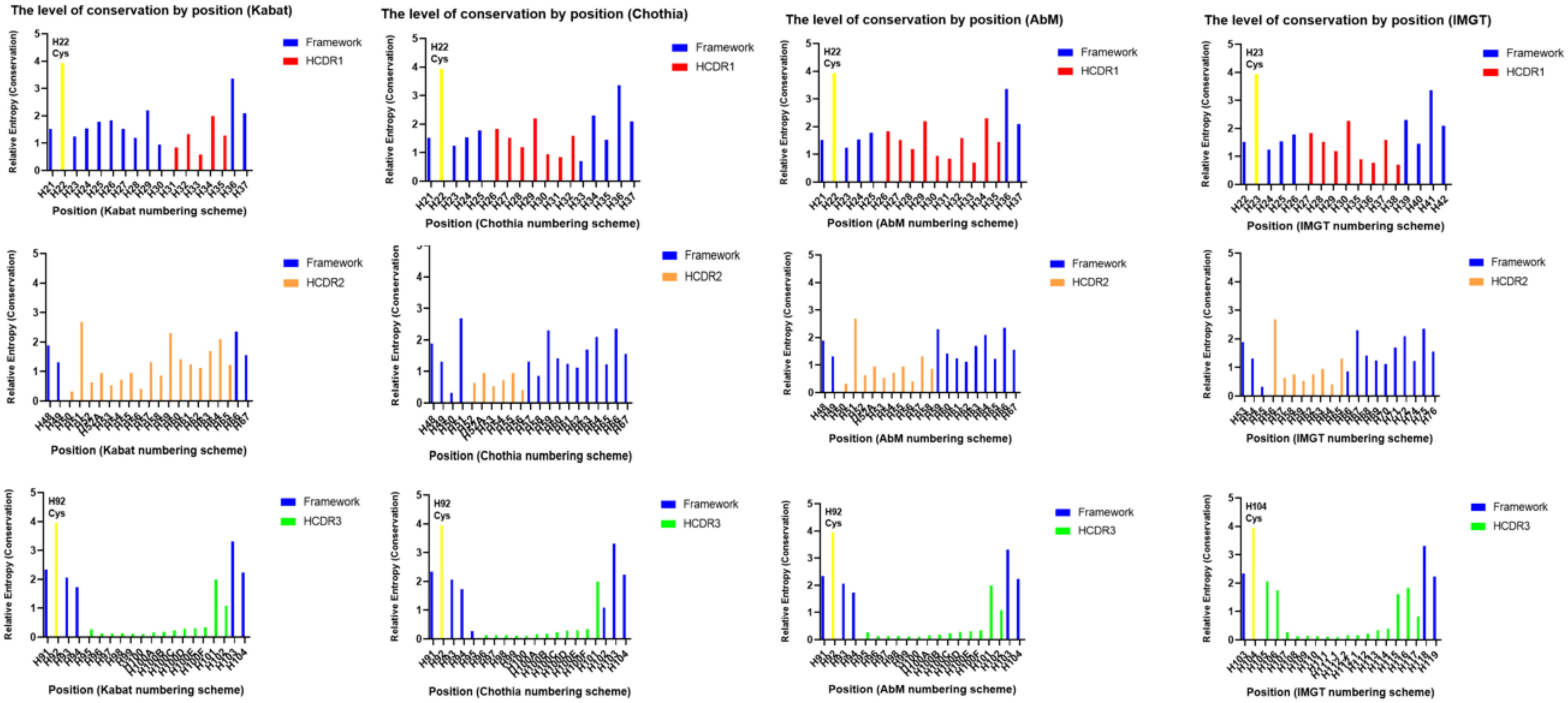

Supplemental Figure S1b. Conservation for selected residues in the variable heavy domain. The conservation was calculated for each residue as the relative entropy between the positional distribution and the all-position reference state, with residue position given by the Kabat, Chothia, AbM and IMGT numbering schemes. Relative entropies of residues within the CDR are colored according to the HCDR to which they belong (HCDR1 shown in red, HCDR2 in orange, and HCDR3 in green), while framework residues are shown in blue. Highlighted cysteine residues are shown in yellow.

Figure S2a

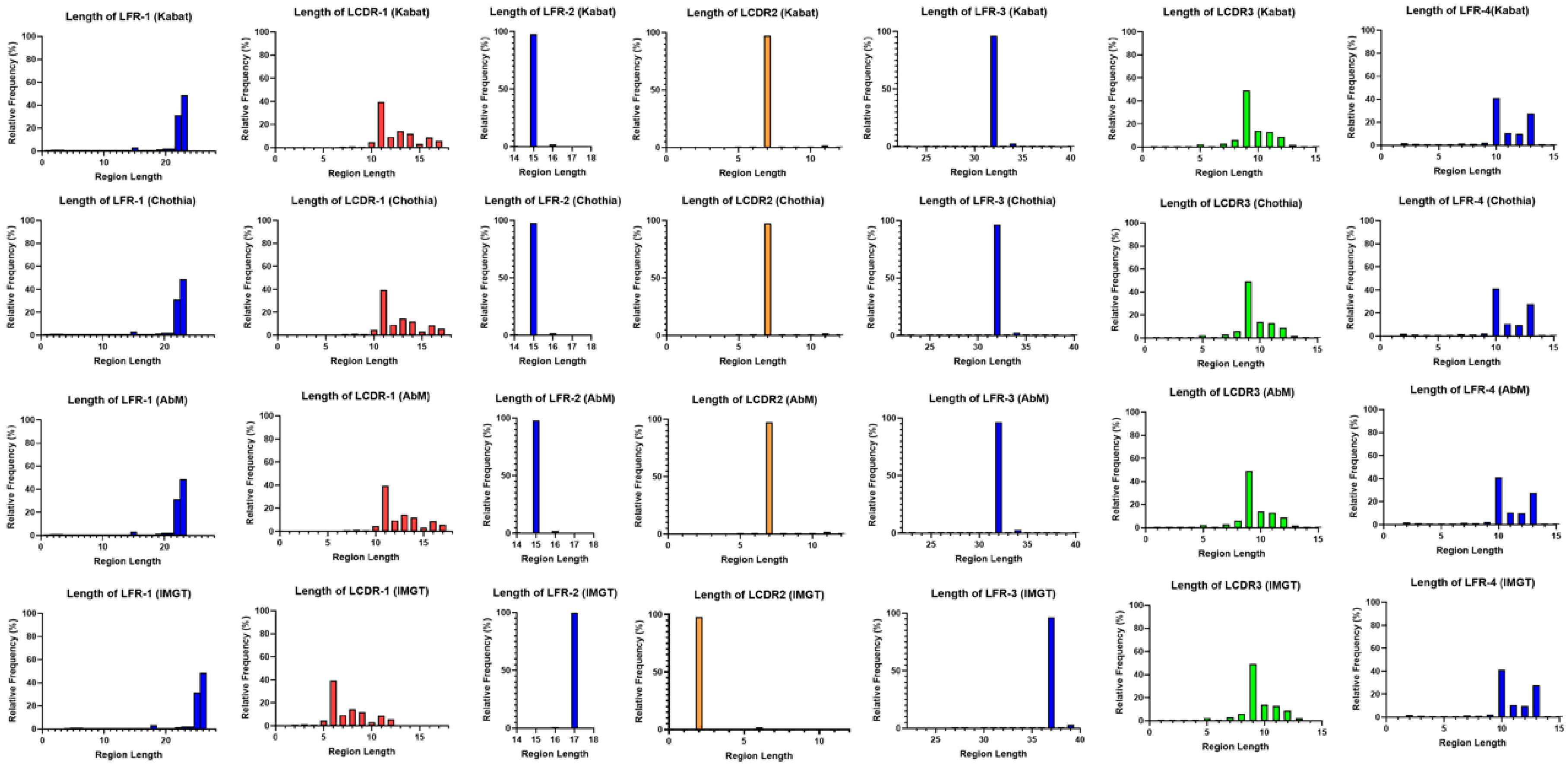

Supplemental Figure S2a. Distribution of LCDR length for numbering schemes. The length distributions of the CDR and framework regions in the variable light domain, as defined by the Kabat, Chothia, AbM and IMGT numbering schemes, are compared.

Figure S2b

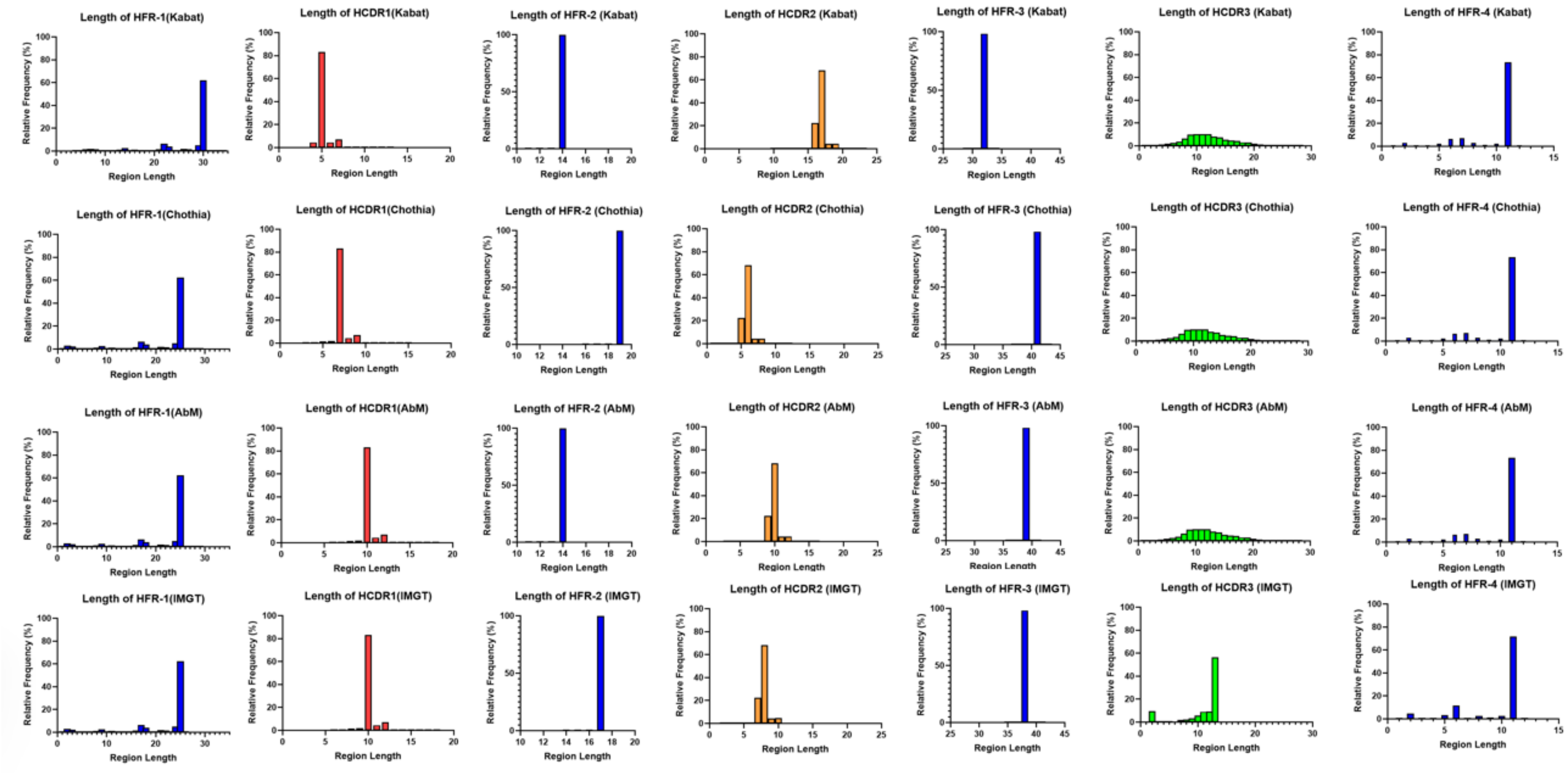

Supplemental Figure S2b. Distribution of HCDRs length for numbering schemes. The length distributions of the CDR and framework regions in the variable heavy domains, as defined by the Kabat, Chothia, AbM and IMGT numbering schemes, are compared.

Figure S3

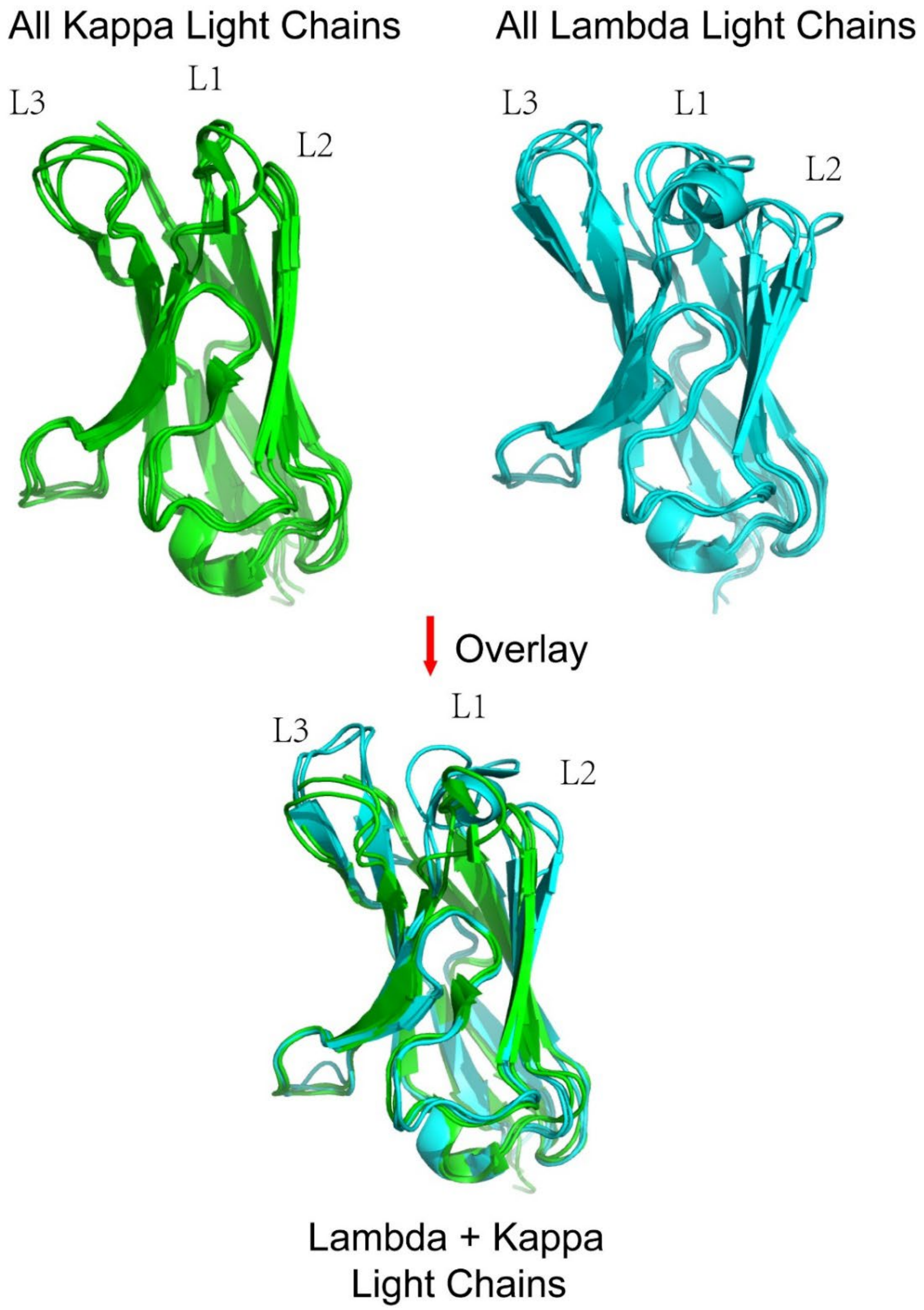

Supplemental Figure S3. Structural comparison of the lambda and kappa light chains. Shown is the structural alignment of the lambda light chain variable domains (Cyan, PDB ID: 6QBC, 5OD0, 4YNY, 6XRJ), the lambda light chain variable domains (Green, PDB ID: 2ZKH, 6TCM, 6Z7X, 5VH3), and the overlay alignment map of the two kappa light chain variable domain and two lambda light variable domain structures (PDB ID: 6QBC, 5OD0, 2ZKH, 6TCM). The lambda light chains exhibit a substantial degree of homology to each other, as do the kappa light chains. However, notably lower levels of homology are observed between the lambda and kappa light chains, particularly within CDR loop regions. The structural alignment was conducted in PyMOL, using the “align” command.

Figure S4

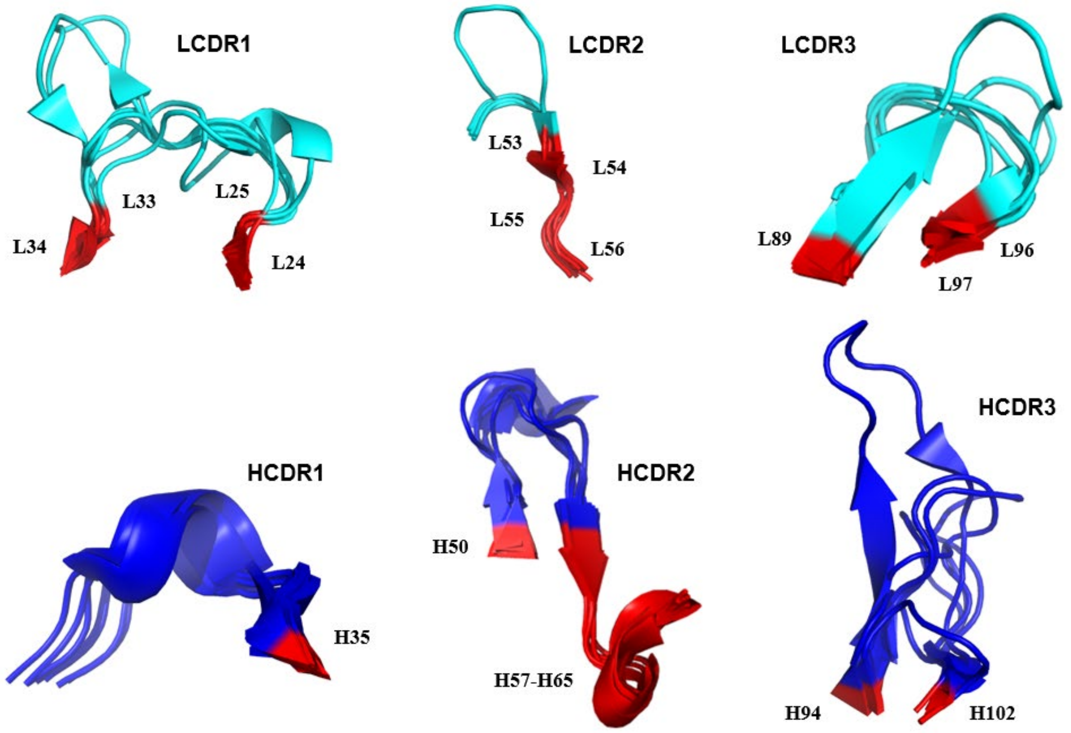

Supplemental Figure S4. Structural alignment of the CDR loops from multiple antibody variable domains. The structural alignment of the light chain variable domains and the heavy chain variable domains (PDB ID: 2ZKH, 6TCM, 6BE2, 6QBC, 6Z7X, 5VH3, 1CR9) are shown. CDR loops were defined according to the "general CDRs", which include all residues defined as CDRs by any numbering scheme and numbered according to the Kabat numbering scheme. Structurally conserved residues are color coded in red and their residue numbers annotated. The structural alignment was conducted in PyMOL, using the “align” command.

Figure S5a

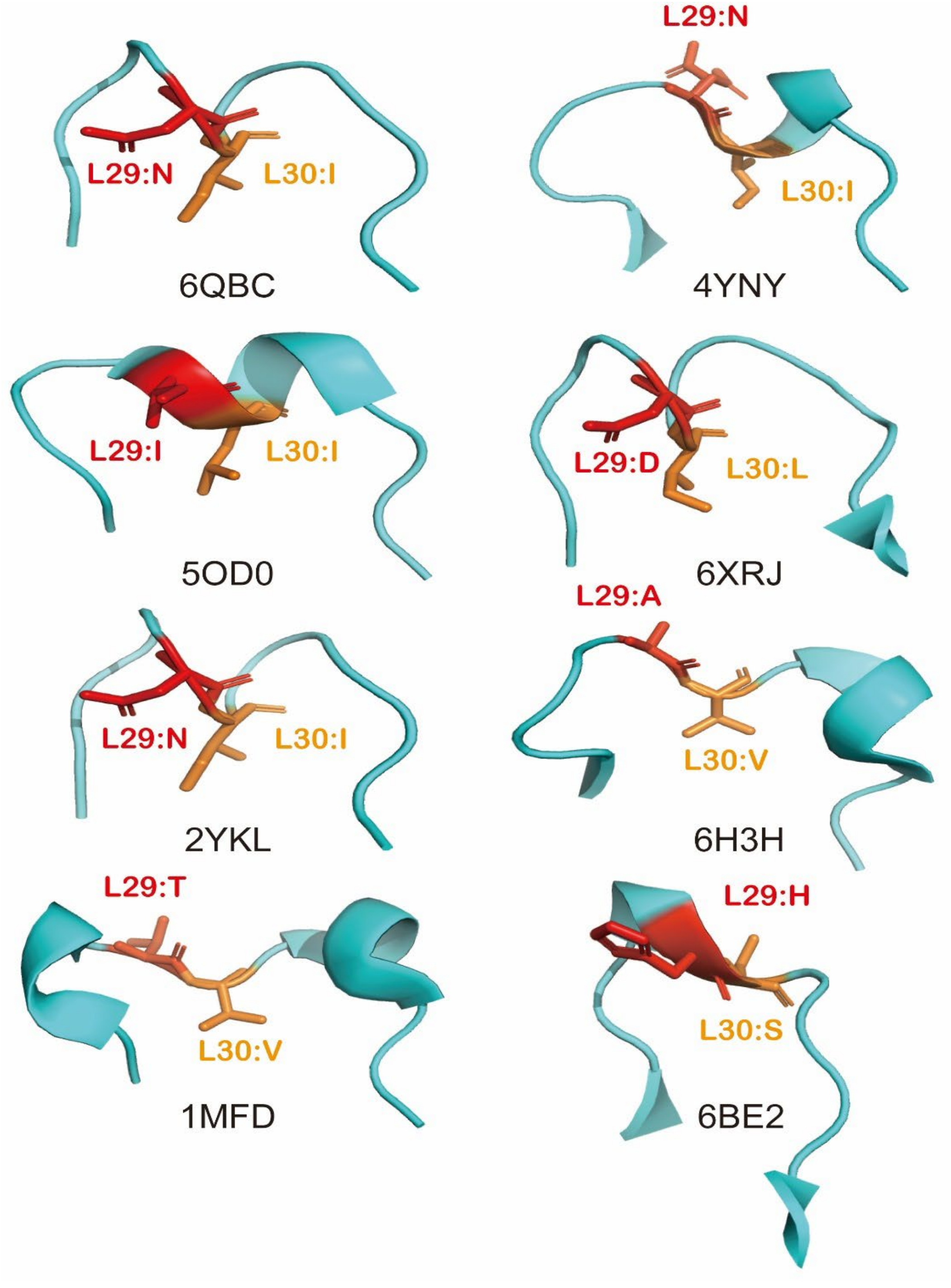

Supplemental Figure S5a. A pivot point in the LCDR1 is located at L30 in the lambda light chain. The LCDR1 loop structure of eight lambda light chain variable domains are shown (PDB ID: 6QBC, 4YNY, 5OD0, 6XRJ, 2YKL, 6H3H, 1MFD, 6BE2). The L29 (red) and L30 (orange) residues (IMGT numbering scheme) are annotated. Note that 6BE2 structure does not show an obvious pivot point feature in the loop—this may be because it was derived from an IgG2 variable domain.

Figure S5b

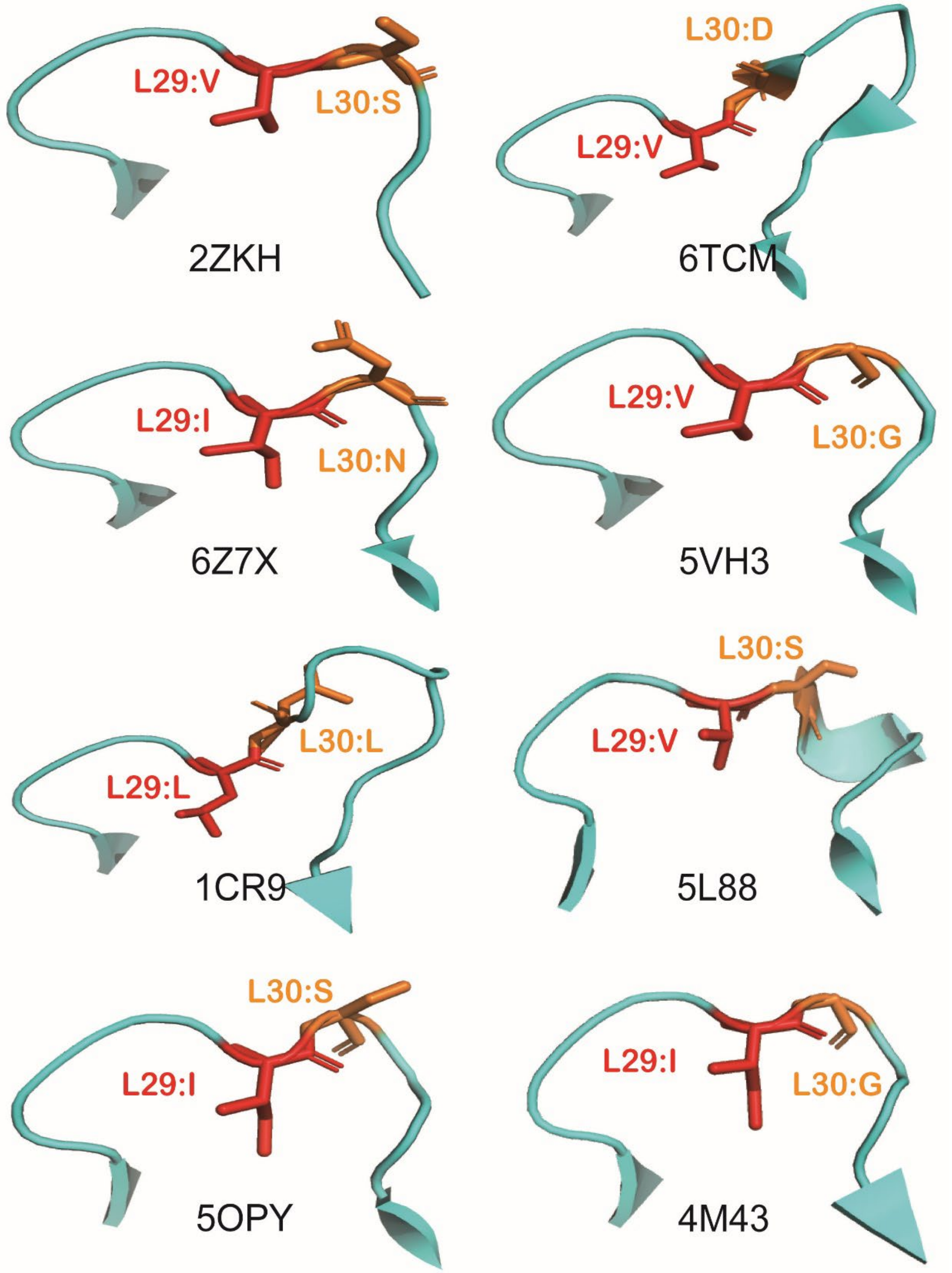

Supplemental Figure S5b. Another pivot point in the LCDR1 is located at L29 in the kappa light chain. The LCDR1 loop structure of eight kappa light chain variable domains are shown (PDB ID: 2ZKH, 6TCM, 6Z7X, 5VH3, 1CR9, 5L88, 5OPY, 4M43). The L29 (red) and L30 (orange) residues (IMGT numbering scheme) are annotated.

Figure S6

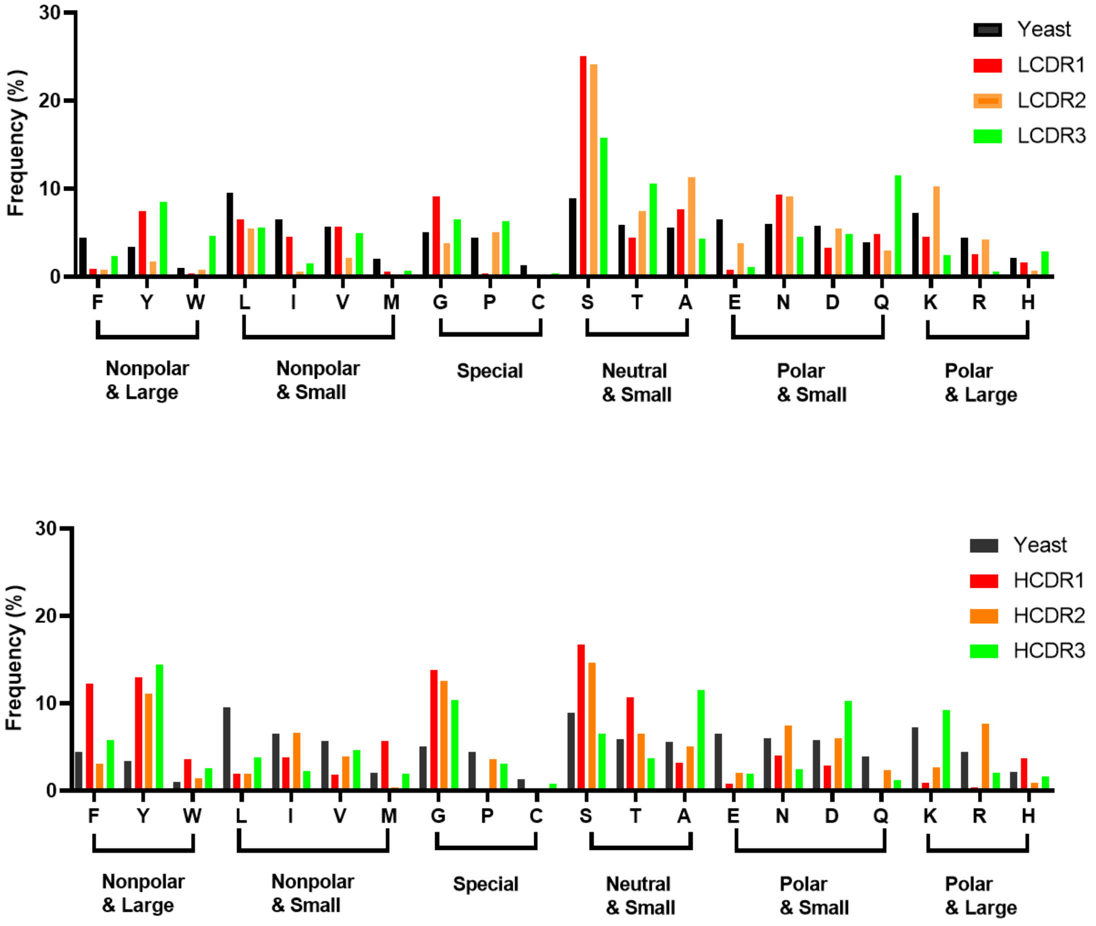

Supplemental Figure S6. Amino acid distribution in each CDR loop compared to reference distribution in yeast. CDR loops were defined according to the "general CDRs", which include all residues defined as CDRs by any numbering scheme (Kabat, Chothia, AbM and IMGT). The reference distribution "yeast" was generated based on codon usage in the yeast proteome.

Figure S7

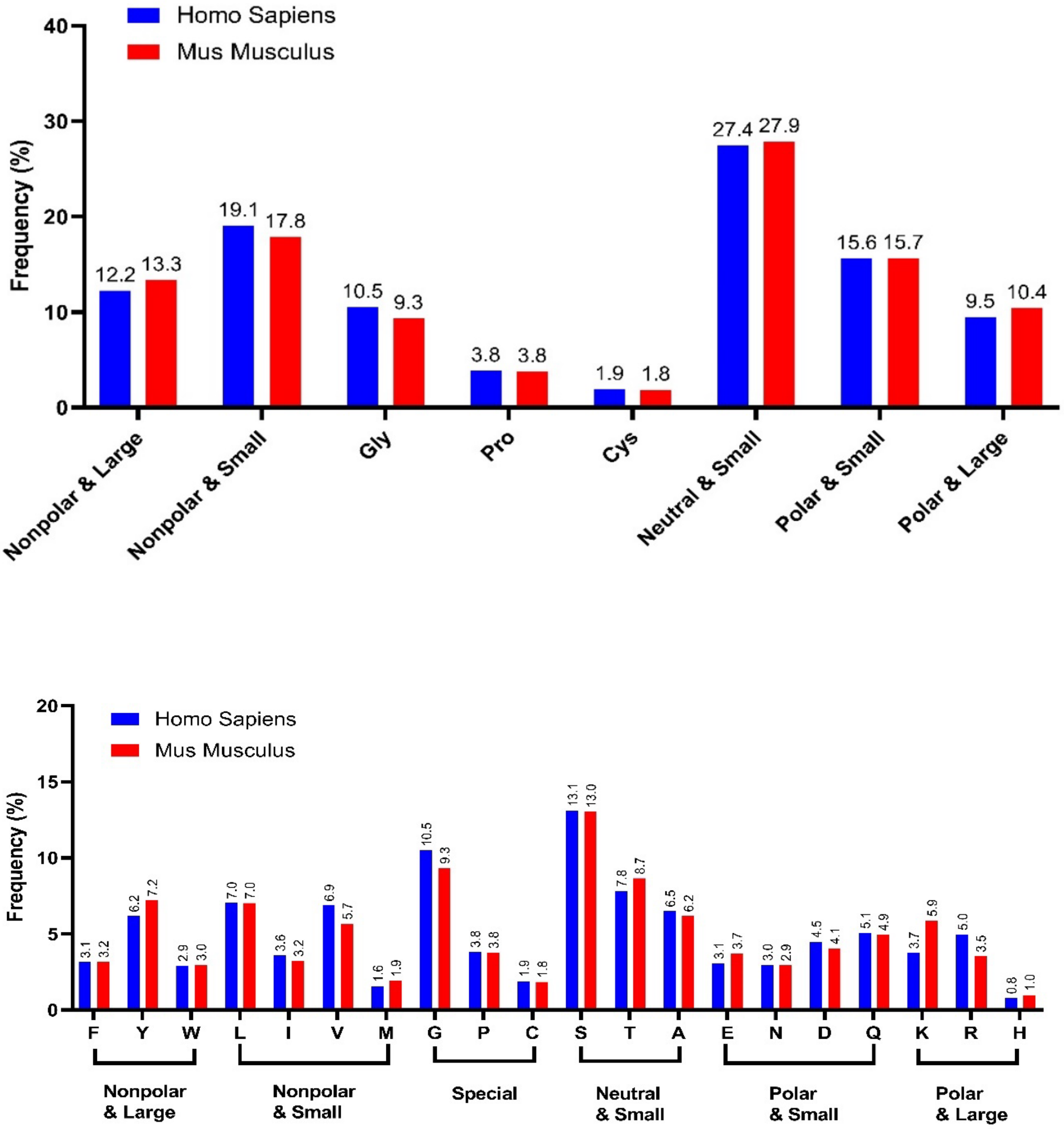

Supplemental Figure S7. The amino acid distribution is similar for both mouse and human antibodies. The amino acid distribution in the mouse and human antibody variable domains are compared, with amino acids grouped based on side chain volume and polarity.

Figure S8

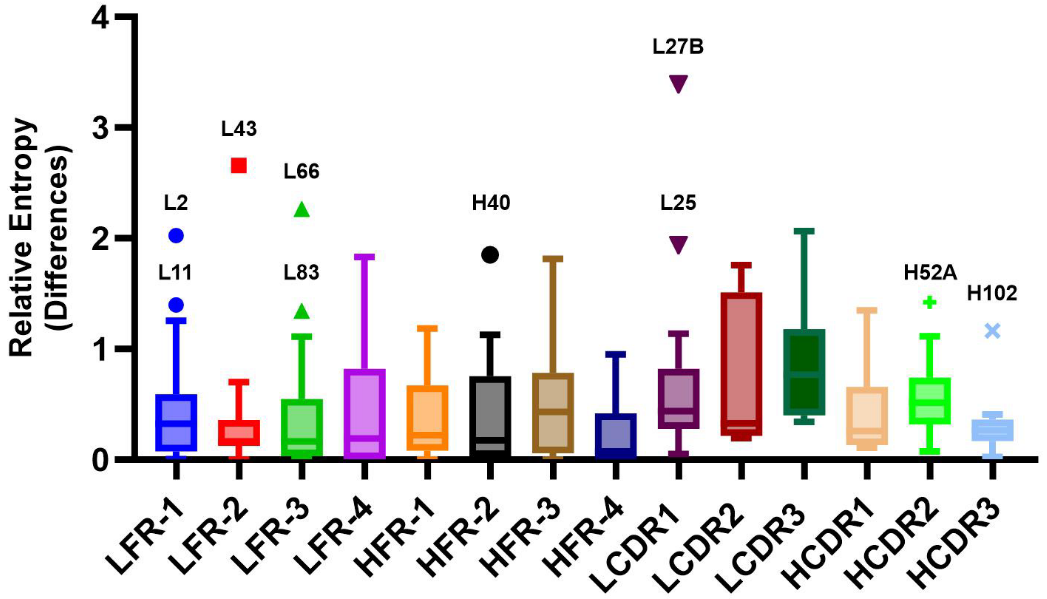

Supplemental Figure S8. Box-and-whisker plot of the regional relative entropy differences between mouse and human variable domains. CDRs are defined based on the inclusive "general CDR" definition and numbered in Kabat numbering scheme. Tukey boxplot was used to generate the plot, which calculates the inter-quartile distance (IQS, the difference between the 25th and 75th percentiles). The value between the 25th and 75th have been drawn as the box. The median is shown as a line within the box. Any data point falling beyond the 75th percentile plus 1.5 times the IQS was represented as an individual point on the plot, and the corresponding residue numbers were annotated above these points for reference.

Figure S9

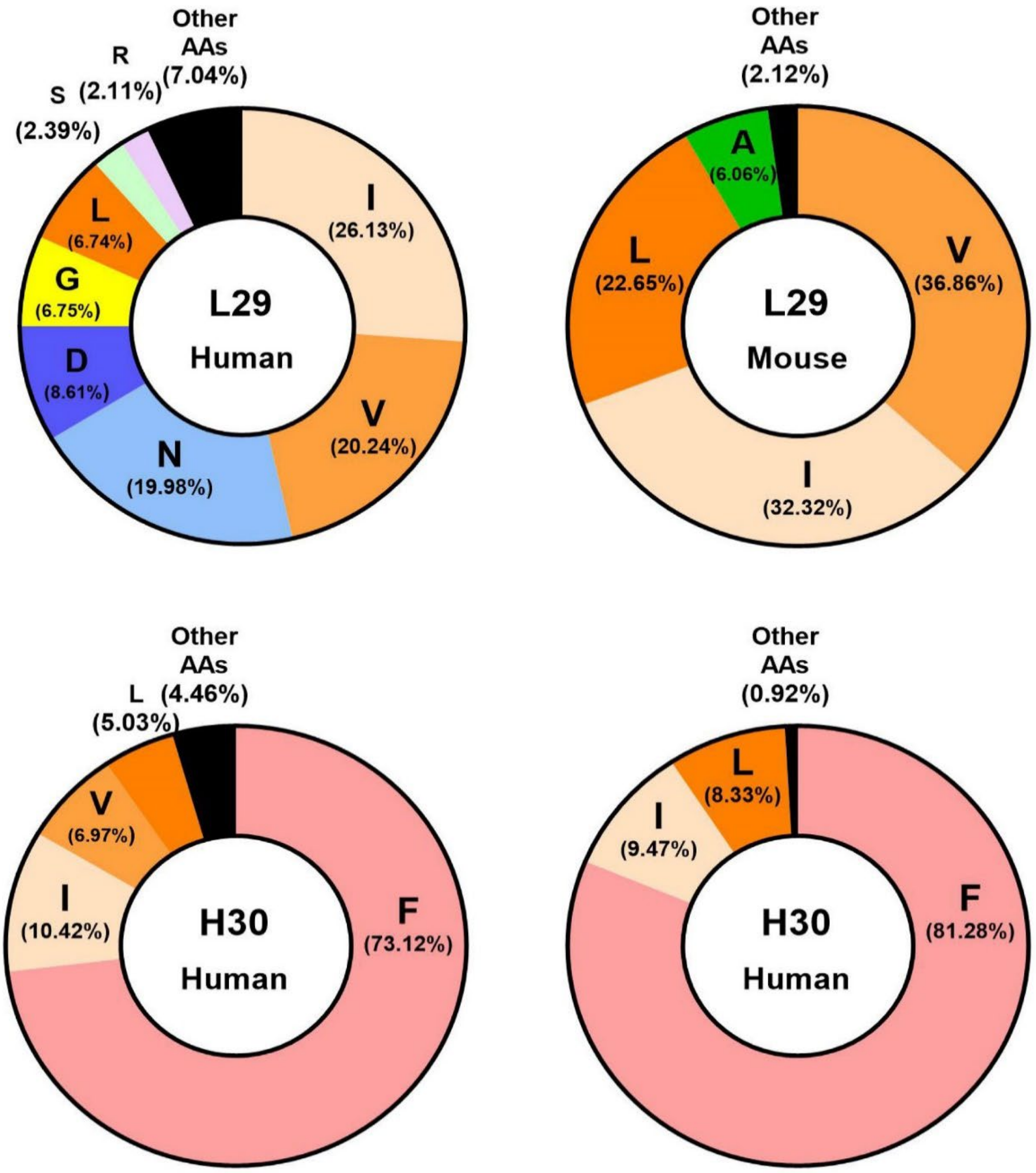

Supplemental Figure S9. Amino acids distribution at pivot points in mouse and human antibody variable domains. The amino acid distributions at the pivot points residues L29 and H30 (IMGT numbering scheme) are shown. Only amino acids exceeding a 2% occupancy rate are explicitly marked, while the frequencies of all other amino acids have been compiled and collectively represented as "Other AAs" in the figures for clarity.

Figure S10

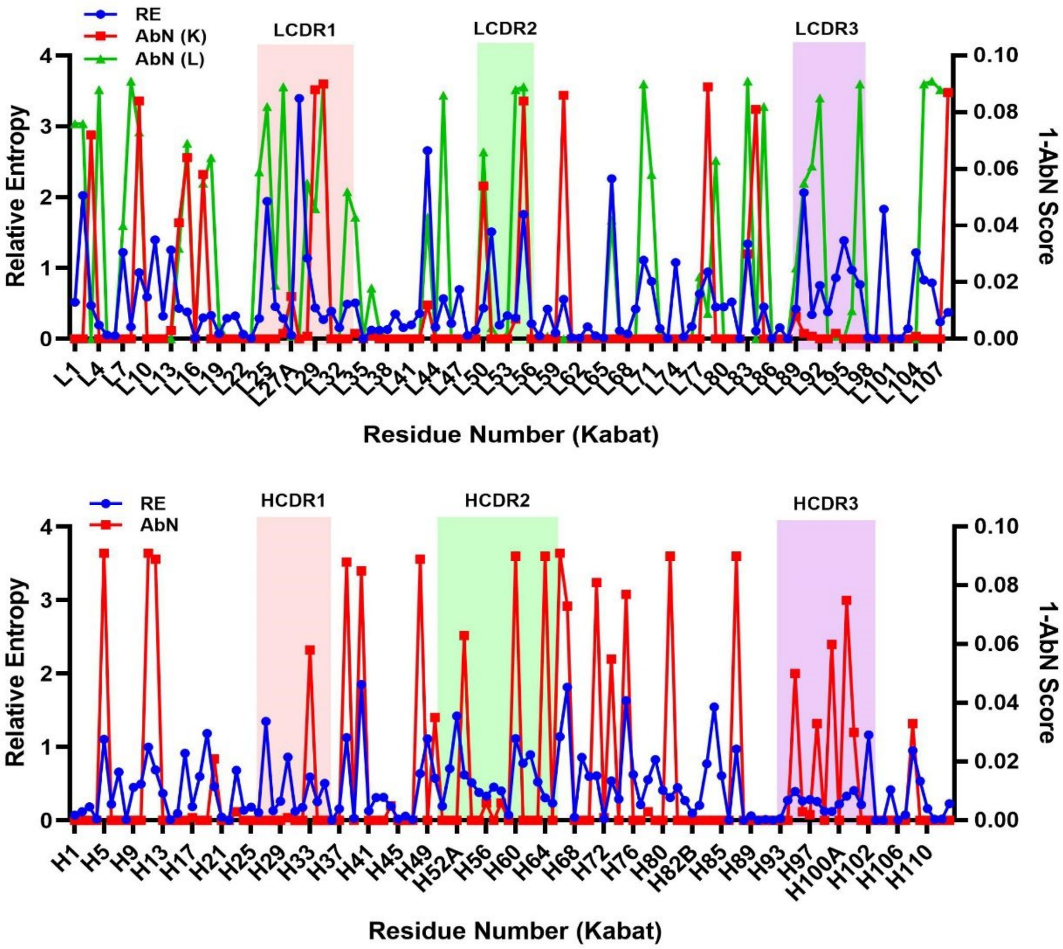

Supplemental Figure S10. The residue-wise relative entropy and the AbNatiV Score calculated based on the consensus mouse sequence in the variable domain. The value of RE and (1-AbNatiV score) of each residue in the variable light (top) and variable heavy (bottom) are shown in the graph. The relative entropy of differences between mouse and human species were shown in blue dots. The AbNatiV score of humanness were shown in red dots or green dots. In the variable light, the AvNatiV score were calculated based on the Variable light Kappa (red dots) and Variable light lambda (green dots) models. To align with the Relative entropy, the AbNatiV score were shown in (1-AbNatiV), which depict the non-humanness of the consensus mouse sequence in each of the position.

Figure S11a

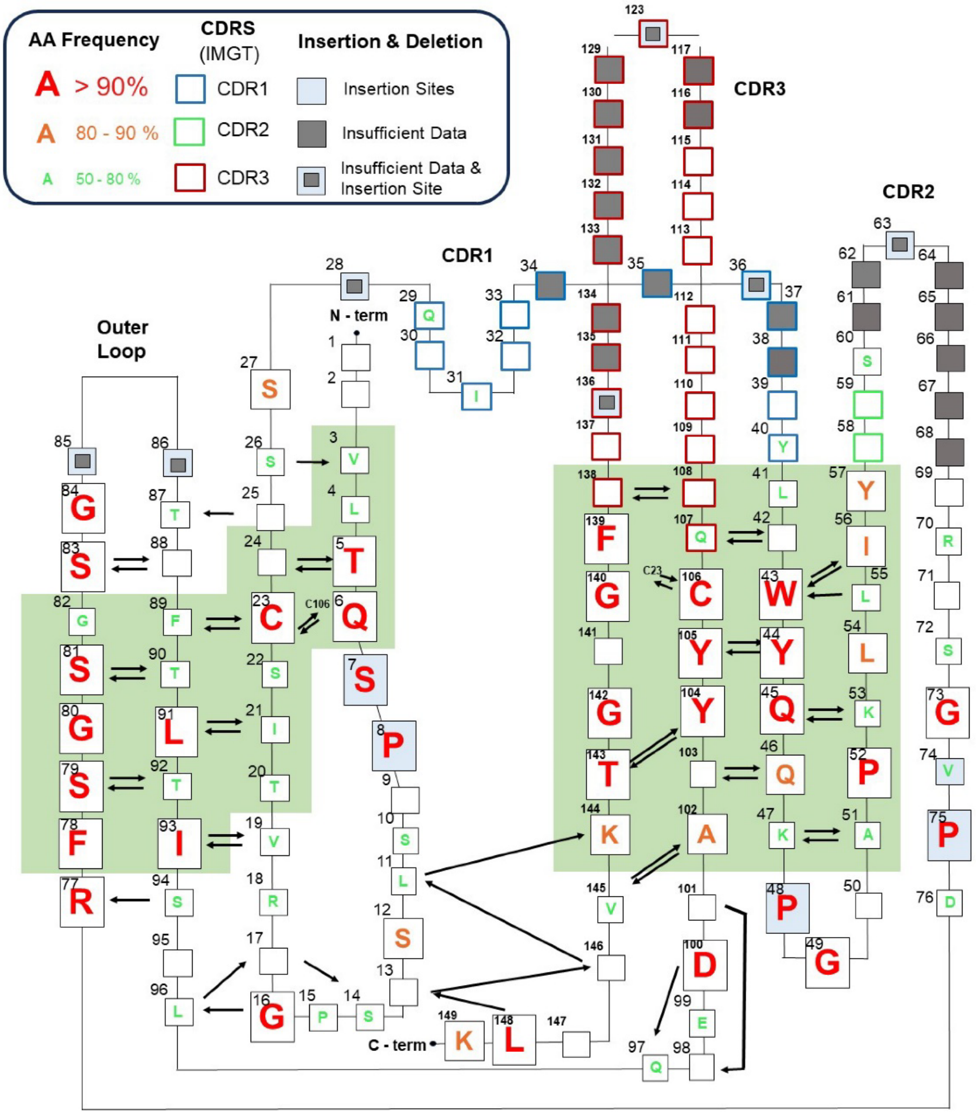

Supplemental Figure S11a. A representation of the consensus sequence, consensus structure, and main-chain hydrogen bonding pattern of human immunoglobulin variable light domains. A 2D map of the variable domain was generated based on the Honnegger numbering scheme. The residues were numbered based on the Aho numbering schemes. Complementarity Determining Regions (CDRs) are defined as per the IMGT numbering scheme. In the map, one-letter codes highlighted in red, orange, and green correspond to amino acid frequencies above 90%, within the range of 80-90%, and between 50% and 80% respectively. CDR residues are marked based on border color, with CDR1 in blue, CDR2 in green, and CDR3 in red. Arrows indicate hydrogen bonds present in the majority of structures for all types of immunoglobulin variable domains. The loop and turn regions which accommodate gaps are indicated in gray. Green areas underlie the residues with Cα positions that are highly structurally conserved.

Figure S11b

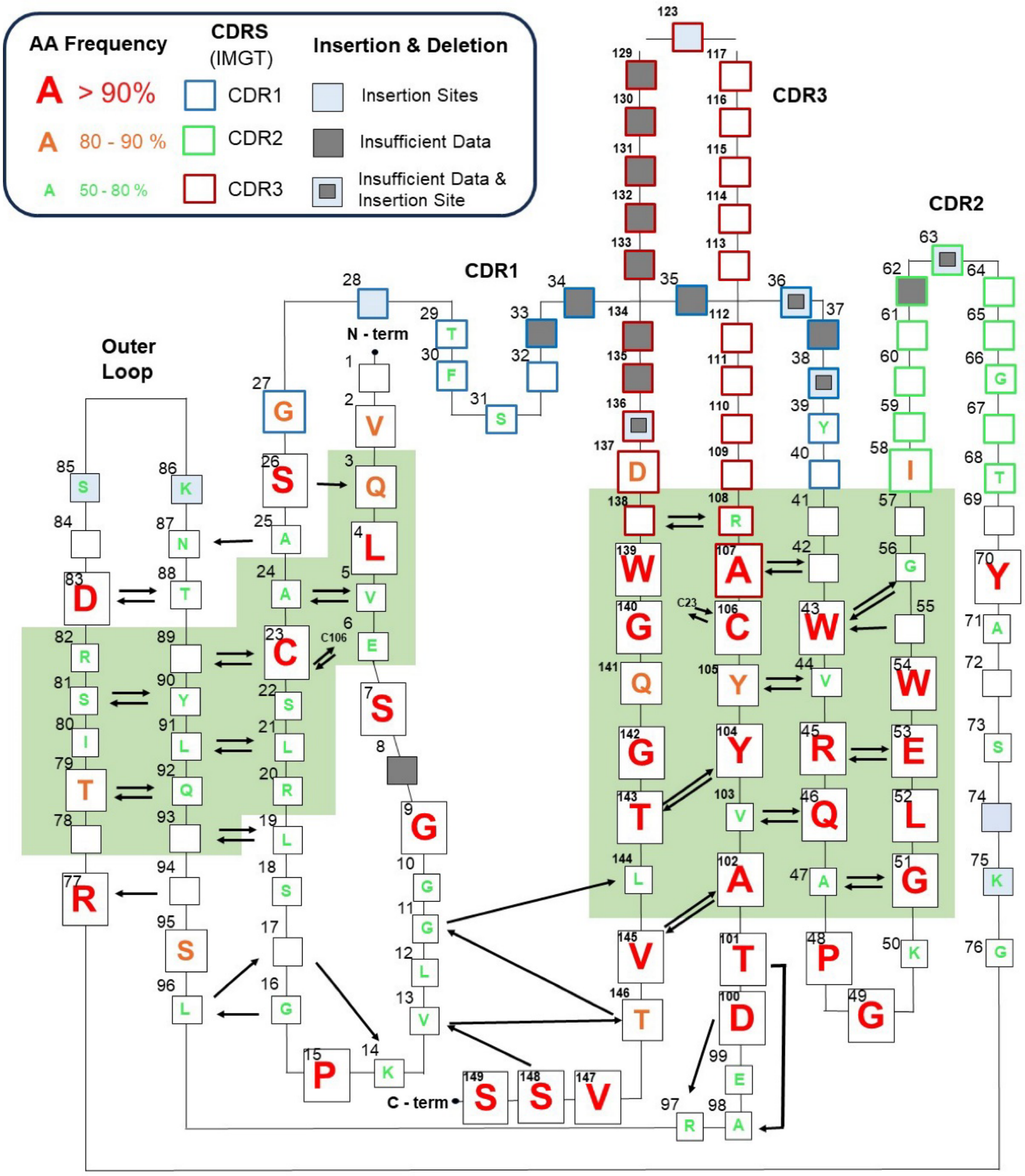

Supplemental Figure S11b. A representation of the consensus sequence, consensus structure, and main-chain hydrogen bonding pattern of human immunoglobulin variable heavy domains. A 2D map of the variable domain was generated based on the Honnegger numbering scheme. The residues were numbered in Aho numbering schemes. Complementarity Determining Regions (CDRs) are defined as per the IMGT numbering scheme. In the map, one-letter codes highlighted in red, orange, and green correspond to amino acid frequencies above 90%, within the range of 80-90%, and between 50% and 80% respectively. CDR residues are marked based on border color, with CDR1 in blue, CDR2 in green, and CDR3 in red. Arrows indicate hydrogen bonds present in the majority of the structures for all types of immunoglobulin variable domains. The loop and turn regions which accommodate gaps are indicated in gray. Green areas underlie the residues with Cα positions that are highly structurally conserved.

SUPPLIEMENTARY TABLE S1

Table of Antibody Variable Domains Amino Acids Distribution and the Codon Usage in Yeast

	Yeast	Variable Domains	Variable Light (VL)	Variable Heavy (VH)	Homo Sapiens Variable Domain	Mus Musculus Variable Domain
A	5.62	6.45	6.11	6.58	6.50	6.18
C	1.29	1.91	1.98	1.89	1.90	1.84
D	5.78	4.32	4.14	4.39	4.49	4.05
E	6.48	3.28	3.04	3.37	3.07	3.72
F	4.45	3.09	3.26	3.03	3.14	3.16
G	5.06	10.26	10.08	10.34	10.49	9.33
H	2.14	0.81	0.81	0.80	0.78	0.98
I	6.51	3.53	4.51	3.15	3.58	3.23
K	7.27	4.29	3.70	4.52	3.74	5.88
L	9.50	7.05	7.22	6.99	7.04	7.02
M	2.09	1.58	0.76	1.90	1.56	1.91
N	6.05	2.97	2.63	3.10	2.97	2.94
P	4.39	3.83	5.71	3.11	3.83	3.77
Q	3.94	5.03	6.36	4.52	5.07	4.94
R	4.42	4.43	3.83	4.66	4.97	3.53
S	8.90	13.14	14.72	12.53	13.09	13.02
T	5.88	8.24	8.78	8.03	7.83	8.65
V	5.65	6.56	5.50	6.98	6.87	5.67
W	1.04	2.84	1.52	3.35	2.92	2.97
Y	3.36	6.34	5.34	6.72	6.17	7.20

Supplemental Table S1. Amino acid frequency calculated based on codon usage (yeast) and sequence stored in the abYsis database (Variable Domains, Variable Light Domain, Variable Heavy Domain, Homo sapiens Variable Domain, Mus musculus Variable Domain).

SUPPLIEMENTARY TABLE S2

Table of Relative Entropy Calculated in each Region in the Variable Domains

Region	Avg. RE (Conservation)	Region	Avg. RE (Conservation)
LFR-1	1.648	HFR-1	1.801
LFR-2	2.177	HFR-2	2.326
LFR-3	2.013	HFR-3	1.891
LFR-4	1.906	HFR-4	2.118
LCDR1	1.035	HCDR1	1.205
LCDR2	1.131	HCDR2	1.204
LCDR3	0.758	HCDR3	0.394
L Framework	1.943	H Framework	1.959
L CDRs	0.923	H CDRs	0.889
Variable Light Domain	1.656	Variable Heavy Domain	1.646
Variable Domains Total	1.651		

Supplemental Table S 2. The average Relative Entropy calculated in each region, with the all-position reference state as the reference state, with CDRs annotated based on the Kabat Numbering Scheme.

SUPPLIEMENTARY TABLE S3

Comparison of the CDR definition of different numbering schemes for immunoglobulin variable domains

LCDR1

Numbering Schemes	*Range*																	
Kabat	24 - 34	24	25	26	27	**27a**	**27b**	**27c**	**27d**	**27e**	**27f**	28	29	30	31	32	33	34
Chothia	26 - 32	24	25	26	27	28	29	30	**30a**	**30b**	**30c**	**30d**	**30e**	**30f**	31	32	33	34
AbM	24 - 34	24	25	26	27	28	29	30	**30a**	**30b**	**30c**	**30d**	**30e**	**30f**	31	32	33	34
IMGT	27 - 38	24	25	26	27	28	29	**30**	**31**	**32**	**33**	**34**	**35**	36	37	38	39	40

LCDR2

Numbering Schemes	*Range*															
Kabat	50 - 56	50	X	X	X	X	X	X	X	X	51	52	53	**54**	55	56
Chothia	50 - 52	50	X	X	X	X	X	X	X	X	51	52	53	**54**	55	56
AbM	50 - 56	50	X	X	X	X	X	X	X	X	51	**52**	53	54	55	56
IMGT	56 - 65	56	**57**	**58**	**59**	**60**	**61**	**62**	**63**	**64**	65	66	67	68	69	70

LCDR3

Numbering Schemes	*Range*																			
Kabat	89 - 97	89	90	91	92	93	94	95	**95a**	**95b**	**95c**	**95d**	**95e**	**95f**	X	X	X	X	96	97
Chothia	91 - 96	89	90	91	92	93	94	95	**95a**	**95b**	**95c**	**95d**	**95e**	**95f**	X	X	X	X	96	97
AbM	89 - 97	89	90	91	92	93	94	95	**95a**	**95b**	**95c**	**95d**	**95e**	**95f**	X	X	X	X	96	97
IMGT	105 - 117	105	106	107	108	109	**110**	**111**	**111.1**	**111.2**	**111.3**	**111.4**	**111.5**	**111.6**	**112**	**113**	114	115	116	117

HCDR1

Numbering Schemes	*Range*																		
Kabat	31 - 35	26	27	28	29	30	31	32	33	34	35	**35a**	**35b**	**35c**	**35d**	**35e**	**35f**	**35g**	**35h**
Chothia	26 - 32	26	27	28	29	30	31	**31a**	**31b**	**31c**	**31d**	**31e**	**31f**	**31g**	**31h**	32	33	34	35
AbM	26 - 35	26	27	28	29	30	31	**31a**	**31b**	**31c**	**31d**	**31e**	**31f**	**31g**	**31h**	32	33	34	35
IMGT	27 - 38	27	28	29	30	**31**	**32**	**32.1**	**32.2**	**32.3**	**32.4**	**33**	**34**	35	36	37	38	39	40

HCDR2

Numbering Schemes	*Range*																				
Kabat	50 - 65	50	51	52	**52a**	**52b**	X	X	53	54	55	56	57	58	59	60	61	62	63	64	65
Chothia	52 - 56	50	51	52	**52a**	**52b**	X	X	53	54	55	56	57	58	59	60	61	62	63	64	65
AbM	50 - 58	50	51	52	**52a**	**52b**	X	X	53	54	55	56	57	58	59	60	61	62	63	64	65
IMGT	56 - 65	55	56	57	58	59	**60***	**61**	62	63	64	65	66	67	68	69	70	71	72	73	74

HCDR3

Numbering Schemes	*Range*																																
Kabat	95 - 102	93	94	95	96	97	98	99	100	**100a**	**100b**	**100c**	**100d**	**100e**	**100f**	**100g**	**100h**	**100i**	**100j**	**100k**	**100l**	**100m**	**100n**	**100o**	**100p**	**100q**	**100r**	**100s**	**100t**	X	X	101	102
Chothia	96 - 101	93	94	95	96	97	98	99	100	**100a**	**100b**	**100c**	**100d**	**100e**	**100f**	**100g**	**100h**	**100i**	**100j**	**100k**	**100l**	**100m**	**100n**	**100o**	**100p**	**100q**	**100r**	**100s**	**100t**	X	X	101	102
AbM	95 - 102	93	94	95	96	97	98	99	100	**100a**	**100b**	**100c**	**100d**	**100e**	**100f**	**100g**	**100h**	**100i**	**100j**	**100k**	**100l**	**100m**	**100n**	**100o**	**100p**	**100q**	**100r**	**100s**	**100t**	X	X	101	102
IMGT	105 - 117	105	106	107	108	109	110	111	**111.1**	**111.2**	**111.3**	**111.4**	**111.5**	**111.6**	**111.7**	**111.8**	**111.9**	**111.10**	**112.9**	**112.8**	**112.7**	**112.6**	**112.5**	**112.4**	**112.3**	**112.2**	**112.1**	112	113	114	115	116	117

Supplemental Table S3. The different numbering schemes for immunoglobulin variable domains (Kabat, Chothia, AbM and IMGT) have been aligned (15, 17, 20, 21, 23, 34). CDR residues were defined based on previously published literature and highlighted within red frames (15, 17, 19, 20, 23). It's worth noting that "Chothia" in this context refers to the Consensus Chothia CDR definition developed by the Martin group (19). Inserted residues have been denoted in **bold** and appear in **blue** text. Residues that lack equivalent counterparts in the respective numbering scheme are indicated by an orange cross " **X**" in the table.

SUPPLIEMENTARY TABLE S4a

Table of Antibody Template PDB Entries, Organism, Light Chain Type and Reference Information

PDB ID	Organism	Light Chain Type	Reference
Structure Alignment Analysis & Pivot Point Sequential Analysis			
2ZKH	Homo Sapiens	**Kappa**	[71]
6TCM	Homo Sapiens	**Kappa**	[72]
6BE2	Homo Sapiens	***Lambda***	[86]
6QBC	Homo Sapiens	***Lambda***	[79]
6Z7X	Mus Musculus	**Kappa**	[73]
5VH3	Mus Musculus	**Kappa**	[74]
1CR9	Mus Musculus	**Kappa**	[75]
Pivot Point Sequential Analysis			
4YNY	Rattus norvegicus	***Lambda***	[80]
5OD0	Homo Sapiens	***Lambda***	[81]
6XRJ	Homo Sapiens	***Lambda***	[82]
2YKL	Homo Sapiens	***Lambda***	[83]
6H3H	Mus Musculus	***Lambda***	[84]
1MFD	Mus Musculus	***Lambda***	[85]
5L88	Mus Musculus	**Kappa**	[76]
5OPY	Mus Musculus	**Kappa**	[77]
4M43	Mus Musculus	**Kappa**	[78]

Supplemental Table S4a. The PDB ID, organism and light chain type information of the immunoglobulin used for structural alignment.

SUPPLIEMENTARY TABLE S4b

Table of Antibody Template Protein Amino Acid Sequences

PDB ID	Sequence
2ZKH	**Light Chain**<br>QVVLTQSPGIMSASPGEKVTITCSASSSVSYMYWFQQKPGTSPKLWIYSTSNLASGVPARFR<br>GSGSGTSYSLTISRMEAEDAATYYCQQRSGYPRTFGGGTKLEIKRA<br>*DAAPTVSIFPPSSEQLTSGGASVVCFLNNFYPKDINVKWKIDGSERQNGVLNSWTDQDSKD*<br>*STYSMSSTLTLTKDEYERHNSYTCEATHKTSTSPIVKSFNRNEC*<br>**Heavy Chain**<br>EVKLEESGGGLVQPGGSMKLSCAASGFTFSDAWMDWVRQSPEKGLEWVAEIRSKVNNHAI<br>HYAESVKGRFTVSRDDSKSSVYLQMNSLRAEDTGIYYCSGWSFLYWGQGTLVTVSA<br>*AKTTPPSVYPLAPGSAAQTNSMVTLGCLVKGYFPEPVTVTWNSGSLSSGVHTFPAVLQSDL*<br>*YTLSSSVTVPSSTWPSETVTCNVAHPASSTKVDKKIVPRD*
6TCM	**Light Chain**<br>DIQLTQSPSSLSASVGDRVTITCRASQSVDYDGDSYMNWYQQKPGKAPKLLIYAASYLESGV<br>PSRFSGSGSGTDFTLTISSLQPEDFATYYCQQSHEDPYTFGQGTKVEIKRTV<br>*AAPSVFIFPPSDEQLKSGTASVVCLLNNFYPREAKVQWKVDNALQSGNSQESVTEQDSKDS*<br>*TYSLSSTLTLSKADYEKHKVYACEVTHQGLSSPVTKSFNRGEC*<br>**Heavy Chain**<br>EVQLVESGGGLVQPGGSLRLSCAVSGYSITSGYSWNWIRQAPGKGLEWVASITYDGSTNYN<br>PSVKGRITISRDDSKNTFYLQMNSLRAEDTAVYYCARGSHYFGHWHFAVWGQGTLVTVSS<br>*ASTKGPSVFPLAPSSKSTSGGTAALGCLVKDYFPEPVTVSWNSGALTSGVHTFPAVLQSSGL*<br>*YSLSSVVTVPSSSLGTQTYICNVNHKPSNTKVDKKVEPKSCHHHHHH*
6BE2	**Light Chain**<br>QLVLTQSPSASASLGASVKLTCTLSSGHSNYAIAWHQQQPGKGPRYLMKVNRDGSHIRGD<br>GIPDRFSGSTSGAERYLTISSLQSEDEADYYCQTWGAGIRVFGGGTKLTVLGQP<br>*KAAPSVTLFPPSSEELQANKATLVCLISDFYPGAVTVAWKADGSPVKAGVETTTPSKQSNNK*<br>*YAASSYLSLTPEQWKSHRSYSCQVTHEGSTVEKTVAPTECS*<br>**Heavy Chain**<br>QVQLQESGPGLVKPSETLSLTCTVSGGSISGYYWSWIRQPPGKGLEWIGYIHYSRSTNSNPAL<br>KSRVTISSDTSKNQLSLRLSSVTAADTAVYYCARDTYYYDSGDYEDAFDIWGQGTMVTVSS<br>*ASTKGPSVFPLAPSSKSTSGGTAALGCLVKDYFPEPVTVSWNSGALTSGVHTFPAVLQSSGL*<br>*YSLSSVVTVPSSSLGTQTYICNVNHKPSNTKVDKKAEPKSC*
6QBC	**Light Chain**<br>QSVLTQPPSASGTPGQRVTISCSGSSSNIGSNTVNWYQQLPGTAPKLLIYSNNQRPSGVPD<br>RFSGSKSGTSASLAISGLQSEDEADYYCAAWDDSLNAWVFGGGTKLTVLGES<br>*EGQPKSSPSVTLFPPSSEELETNKATLVCTITDFYPGVVTVDWKVDGTPVTQGMETTQPSKQ*<br>*SNNKYMASSYLTLTARAWERHSSYSCQVTHEGHTVEKSLSRADCS*<br>**Heavy Chain**<br>QVTLKESGGGLVKPGGSLRLSCAASGFTFSSYSMNWVRQAPGKGLEWVSSISSSSSYIYYAD<br>SVKGRFTISRDNAKNSLYLQMNSLRAEDTAVYYCARQVGATWAFDIWGQGTLVTVSA<br>*AKTTPPSVYPLAPGSAAQTNSMVTLGCLVKGYFPEPVTVTWNSGSLSSGVHTFPAVLQSDL*<br>*YTLSSSVTVPSSPRPSETVTCNVAHPASSTKVDKKIVPRDCAAAENLYFQ*
6Z7X	**Light Chain**<br>DIQMTQSPSSLSASLGGRVTITCKASQDINKYLAWYQHKPGKGPRLLIHYTSTLQPGIPSRFS<br>GSGSGRDYSFSISNLEPEDVATYYCLQYDSLLSFGAGTKLELKRA<br>*DAAPTVSIFPPSSEQLTSGGASVVCFLNNFYPKDINVKWKIDGSERQNGVLNSWTDQDSKD*<br>*STYSMSSTLTLTKDEYERHNSYTCEATHKTSTSPIVKSFNRNEC*<br>**Heavy Chain**<br>EVQLVESGGGLVKPGGSLKLSCTASGFAFSDYDMSWVRQTPEKRLEWVAFISNGGYSTYYP

	DTVKGRFTISRDNAENTLYLQMSSLKSEDTAIYYCARQGLRYFDYWGLGTTLTVSS<br>*AKTTPPSVYPLAPGSAAQTNSMVTLGCLVKGYFPEPVTVTWNSGSLSSGVHTFPAVLQSDL YTLSSSVTVPSSTWPSETVTCNVAHPASSTKVDKKIVPRDCG*
5VH3	**Light Chain**<br>DILLTQSPAILSVSPGERVSFSCRASQFVGSSIHWYQQRTNGSPRLLIKYASESMSGIPSRFSGS GSGTDFTLSINTVESEDIADYYCQQSHSWPFTFGSGTNLEVKRTV<br>*AAPSVFIFPPSDEQLKSGTASVVCLLNNFYPREAKVQWKVDNALQSGNSQESVTEQDSKDS TYSLSSTLTLSKADYEKHKVYACEVTHQGLSSPVTKSFNRGEC*<br>**Heavy Chain**<br>EVKLEESGGGLVQPGGSMKLSCVASGFIFSNHWMNWVRQSPEKGLEWVAEIRSKSINSATH YAESVKGRFTISRDDSKSAVYLQMTDLRTEDTGVYYCSRNYYGSTYDYWGQGTTLTVSS<br>*ASTKGPSVFPLAPSSKSTSGGTAALGCLVKDYFPEPVTVSWNSGALTSGVHTFPAVLQSSGL YSLSSVVTVPSSSLGTQTYICNVNHKPSNTKVDKKVEPKSCDKT*
1CR9	**Light Chain**<br>DVVMTQTPLSLSVTIGQPASISCKSSQSLLDSDGKTYLIWVFQRPGQSPKRLIFLVSKRDSGVP DRFTGSGSGTDFTLKISRVEAEDVGVYYCWQGTHFPHTVGGGTKLEIARAD<br>*AAPTVSIFPPSSEQLTSGGASVVCFLNNFYPKDINVKWKIDGSERQNGVLNSWTDQDSKDS TYSMSSTLTLTKDEYERHNSYTCEATHKTSTSPIVKSFNRNEC*<br>**Heavy Chain**<br>KVKLQQSGAELVRSGASVKLSCTASGFNIKDYYIQWVKQRPEQGLEWIGWIDPENGNSEYA PRFQGKATMTADTLSNTAYLQLSSLTSEDTAVYYCNADLHDYWGQGTTLTVSS<br>*AKTTAPSVYPLAPVCGDTTGSSVTLGCLVKGYFPEPVTLTWNSGSLSSGVHTFPAVLQSDLYT LSSSVTVTSSTWPSQSITCNVAHPASSTKVDKKIEPRVTS*

Supplemental Table S4b. The amino acid sequences of the immunoglobulin used in the structure alignment. Sequences belonging to constant domains are *italicized*.

SUPPLIEMENTARY TABLE S5a

Table of Antibody Template Protein LCDRs Amino Acid Sequences

PDB ID	Numbering schemes	LCDR1	LCDR2	LCDR3
2ZKH	Kabat	SASSSVSYMY	STSNLAS	QQRSGYPRT
	Chothia	SSSVSY	STS	RSGYPR
	AbM	SASSSVSYMY	STSNLAS	QQRSGYPRT
	IMGT	SSVSY	ST	QQRSGYPRT
6TCM	Kabat	RASQSVDYDGDSYMN	AASYLES	QQSHEDPYT
	Chothia	SQSVDYDGDSY	AAS	SHEDPY
	AbM	RASQSVDYDGDSYMN	AASYLES	QQSHEDPYT
	IMGT	QSVDYDGDSY	AA	QQSHEDPYT
6BE2	Kabat	TLSSGHSNYAIA	VNRDGSHIRGD	QTWGAGIRV
	Chothia	SSGHSNYA	VNR	WGAGIR
	AbM	TLSSGHSNYAIA	VNRDGSHIRGD	QTWGAGIRV
	IMGT	SGHSNYA	VNRDGS	QTWGAGIRV
6QBC	Kabat	SGSSSNIGSNTVN	SNNQRPS	AAWDDSLNAWV
	Chothia	SSSNIGSNT	SNN	WDDSLNAW
	AbM	SGSSSNIGSNTVN	SNNQRPS	AAWDDSLNAWV
	IMGT	SSNIGSNT	SN	AAWDDSLNAWV
6Z7X	Kabat	KASQDINKYLA	YTSTLQP	LQYDSLLS
	Chothia	SQDINKY	YTS	YDSLL
	AbM	KASQDINKYLA	YTSTLQP	LQYDSLLS
	IMGT	QDINKY	YT	LQYDSLLS
5VH3	Kabat	RASQFVGSSIH	YASESMS	QQSHSWPFT
	Chothia	SQFVGSS	YAS	SHSWPF
	AbM	RASQFVGSSIH	YASESMS	QQSHSWPFT
	IMGT	QFVGSS	YA	QQSHSWPFT
1CR9	Kabat	KSSQSLLDSDGKTYLI	LVSKRDS	WQGTHFPHT
	Chothia	SQSLLDSDGKTY	LVS	GTHFPH
	AbM	KSSQSLLDSDGKTYLI	LVSKRDS	WQGTHFPHT
	IMGT	QSLLDSDGKTY	LV	WQGTHFPHT

Supplemental Table S5a. The amino acid sequences of the LCDRs predicted by the Kabat, Chothia, AbM (Martin) and IMGT numbering schemes.

SUPPLIEMENTARY TABLE S5b

Table of Antibody Template Protein HCDRs Amino Acid Sequences

PDB ID	Numbering schemes	HCDR1	HCDR2	HCDR3
2ZKH	Kabat	DAWMD	EIRSKVNNHAIHYAESVKG	WSFLY
	Chothia	GFTFSDA	RSKVNNHA	WSFLY
	AbM	GFTFSDAWMD	EIRSKVNNHAIH	WSFLY
	IMGT	GFTFSDAW	IRSKVNNHAI	SGWSFLY
6TCM	Kabat	SGYSWN	SITYDGSTNYNPSVKG	GSHYFGHWHFAV
	Chothia	GYSITSGY	TYDGS	GSHYFGHWHFAV
	AbM	GYSITSGYSWN	SITYDGSTN	GSHYFGHWHFAV
	IMGT	GYSITSGYS	ITYDGST	ARGSHYFGHWHFAV
6BE2	Kabat	GYYWS	YIHYSRSTNSNPALKS	DTYYYDSGDYEDAFDI
	Chothia	GGSISGY	HYSRS	DTYYYDSGDYEDAFDI
	AbM	GGSISGYYWS	YIHYSRSTN	DTYYYDSGDYEDAFDI
	IMGT	GGSISGYY	IHYSRST	ARDTYYYDSGDYEDAFDI
6QBC	Kabat	SYSMN	SISSSSSYIYYADSVKG	QVGATWAFDI
	Chothia	GFTFSSY	SSSSSY	QVGATWAFDI
	AbM	GFTFSSYSMN	SISSSSSYIY	QVGATWAFDI
	IMGT	GFTFSSYS	ISSSSSYI	ARQVGATWAFDI
6Z7X	Kabat	DYDMS	FISNGGYSTYYPDTVKG	QGLRYFDY
	Chothia	GFAFSDY	SNGGYS	QGLRYFDY
	AbM	GFAFSDYDMS	FISNGGYSTY	QGLRYFDY
	IMGT	GFAFSDYD	ISNGGYST	ARQGLRYFDY
5VH3	Kabat	NHWMN	EIRSKSINSATHYAESVKG	NYYGSTYDY
	Chothia	GFIFSNH	RSKSINSA	NYYGSTYDY
	AbM	GFIFSNHWMN	EIRSKSINSATH	NYYGSTYDY
	IMGT	GFIFSNHW	IRSKSINSAT	SRNYYGSTYDY
1CR9	Kabat	DYYIQ	WIDPENGNSEYAPRFQG	DLHDY
	Chothia	GFNIKDY	DPENGN	DLHDY
	AbM	GFNIKDYYIQ	WIDPENGNSE	DLHDY
	IMGT	GFNIKDYY	IDPENGNS	NADLHDY

Supplemental Table S5b. The amino acid sequences of the HCDRs predicted by the Kabat, Chothia, AbM (Martin) and IMGT numbering schemes.

SUPPLIEMENTARY TABLE S6

Table of Sequential and Structural Conserved Residues in CDRs

Loop	Kabat		Chothia		AbM		IMGT	
	Seq.	Str.	Seq.	Str.	Seq.	Str.	Seq.	Str.
LCDR1	-	L24-L25<br>L32-L34	-	-	-	L24-L25<br>L32-L34	-	-
LCDR2	L54	L53-L56	-	L50	L54	L53-L56	-	56
LCDR3	-	L89-L90<br>L97	-	-	-	L89-L90<br>L97	-	-
HCDR1	-	H35	-	-	-	H35	30	-
HCDR2	H51	H50,<br>H57-H65	-	-	H51	H50,<br>H57-H58	56	65
HCDR3	H101	H102	H101	-	H101	H102	105,116	105, 117

Supplemental Table S6. The sequence (Seq.) and structurally (Str.) conserved residues in CDRs identified based on each numbering scheme. To identify sequence conservation, residues with Relative Entropy (RE) values exceeding the average RE for the region by 1.65 times the standard deviation were flagged. Meanwhile, structural conservation was ascertained through structural alignment (see Supplemental Figure 4). Residues were annotated and numbered in accordance with each of the respective numbering schemes.

SUPPLIEMENTARY TABLE S7

Table of Antibody Template Protein Light Chain Amino Acid Sequences

PDB ID	Sequence
**Lambda Light Chains**	
6QBC	QSVLTQPPSASGTPGQRVTISCSGSSS**NI**GSNTVNWYQQLPGTAPKLLIYSNNQRPSGVPDRFSGSKSGTSASLAISGLQSEDEADYYCAAWDDSLNAWVFGGGTKLTVLGES
4YNY	QFVLTQPNSVSTNLGSTVKLSCKRSTG**NI**GSNYVNWYQQHEGRSPTTMIYRDDKRPDGVPDRFSGSIDRSSNSALLTINNVQTEDEADYFCHSYSSGIVFGGGTKLTVLGQP
5OD0	QSVWTQPPSVSAAPGQKVTISCSGDDS**I**LRSAFVSWYQQVPGSAPKLVIFDDRQRPSGIPARFSGSNSGTTATLDIAGLQRGDEADYYCAAWNGRLSAFVFGSGTKLTVLGQP
6XRJ	ALTQPPSVSGSPGQSVIISCTGTSS**DI**GQYNSVSWYQQHPDKAPKLVIYGVTSRPSGVSDRFSGSKYGDTASLTISGLQAEDEADYYCSSHADENMALFGGGTRLTVLGQP
2YKL	QSELTQPPSASGTPGQRVTISCSGSSS**NI**GSNYVYWYQQLPGTAPKLLIYRNNQRPSGVPDRFSGSKSGTSASLAISGLRSEDEADYYCAAWDDSLSAWVFGGGTQLDILGQP
6H3H	QAVVTQESALTTSPGETVTLTCRSSTG**AV**TTSNYANWVQEKPDHLFTGLIGGTNNRAPGVPARFSGSLIGDKAALTITGAQTEDEAIYFCALWYSNHWVFGGGTKLTVLGQP
1MFD	QAVVTQESALTTSPGETVTLTCRSSTG**TV**TSGNHANWVQEKPDHLFTGLIGDTNNRAPGVPARFSGSLIGDKAALTITGAQPEDEAIYFCALWCNNHWIFGGGTKLTVLGQP
**6BE2**	QLVLTQSPSASASLGASVKLTCTLSSG**HS**NYAIAWHQQQPGKGPRYLMKVNRDGSHIRGDGIPDRFSGSTSGAERYLTISSLQSEDEADYYCQTWGAGIRVFGGGTKLTVLGQP
**Kappa Light Chains**	
2ZKH	QVVLTQSPGIMSASPGEKVTITCSASSS**VS**YMYWFQQKPGTSPKLWIYSTSNLASGVPARFRGSGSGTSYSLTISRMEAEDAATYYCQQRSGYPRTFGGGTKLEIKRA
6TCM	DIQLTQSPSSLSASVGDRVTITCRASQS**VD**YDGDSYMNWYQQKPGKAPKLLIYAASYLESGVPSRFSGSGSGTDFTLTISSLQPEDFATYYCQQSHEDPYTFGQGTKVEIKRTV
6Z7X	DIQMTQSPSSLSASLGGRVTITCKASQD**IN**KYLAWYQHKPGKGPRLLIHYTSTLQPGIPSRFSGSGSGRDYSFSISNLEPEDVATYYCLQYDSLLSFGAGTKLELKRA
5VH3	DILLTQSPAILSVSPGERVSFSCRASQF**VG**SSIHWYQQRTNGSPRLLIKYASESMSGIPSRFSGSGSGTDFTLSINTVESEDIADYYCQQSHSWPFTFGSGTNLEVKRTV
1CR9	DVVMTQTPLSLSVTIGQPASISCKSSQS**LL**DSDGKTYLIWVFQRPGQSPKRLIFLVSKRDSGVPDRFTGSGSGTDFTLKISRVEAEDVGVYYCWQGTHFPHTVGGGTKLEIARAD
5L88	DIVLTQTPAIMSASLGERVTMTCTANSS**VS**SNYFHWYQQKPGSSPKLWIYSTSNLASGVPTRFSGSGSGTSYSLTLSSMEAEDAATYYCHQYHRSPPTFGSGTKLKMKRA
5OPY	ELVMTQTPATLSVTPGDSVSLSCRASQS**IS**NHLHWYQQKSHESPRLLIKYASQSISGIPSRFSGSGSGTDFTLSINSVETEDFGMYFCQQSNSWPHTFGGGTKLEIKRA
4M43	ELVMTQSPAILSVSPGERVSFSCRASQI**IG**TSIHWYQQRTNGSPRLLIKYASESISGIPSRFSGSGSGTDFTLTINSVESDDIADYYCQQSNSWPVTFGAGTKLELKRA

Supplemental Table S7. The PDB ID and the amino acid sequences of eight lambda light chains and eight kappa light chains are used for pivot point structural and sequential analysis. The L29 and H30 residues (IMGT numbering scheme) are in **bold** and colored in red. The L30 residues (IMGT numbering scheme) are in **bold** and colored in orange. The 6BE2 sequence is also highlighted because it has no pivot point feature (see Supplemental Figure 5a) . This sequence was later found to originate from a variable domain in IgG2.

SUPPLIEMENTARY TABLE S8a

Table of Relative Entropy of the Residues in LCDR1 based on different numbering schemes

Kabat		Chothia		AbM		IMGT	
L23	3.842	L23	3.842	L23	3.842	23	3.889
L24	1.084	L24	1.084	L24	1.084	24	1.084
L25	1.084	L25	1.084	L25	1.084	25	1.084
L26	1.341	L26	1.341	L26	1.341	26	1.341
L27	1.199	L27	1.199	L27	1.199	27	1.199
L27a	1.264	L28	0.856	L28	0.856	28	0.860
**L27b**	**1.126**	**L29**	**0.938**	**L29**	**0.938**	**29**	**0.984**
L27c	1.075	L30	0.507	L30	0.507	30	0.951
L27d	-	L30a	0.752	L30a	0.752	31	0.994
L27e	-	L30b	0.675	L30b	0.675	32	-
L27f	-	L30c	0.711	L30c	0.711	33	-
L28	0.593	L30d	-	L30d	-	34	-
L29	0.710	L30e	-	L30e	-	35	0.969
L30	0.580	L30f	-	L30f	-	36	0.657
L31	0.980	L31	1.023	L31	1.023	37	0.993
L32	1.158	L32	1.173	L32	1.173	38	1.158
L33	1.389	L33	1.443	L33	1.426	39	1.389
L34	0.940	L34	0.940	L34	0.940	40	0.940
L35	4.141	L35	4.141	L35	4.141	41	3.356

Supplemental Table S8a. The relative entropy of conservation calculated in LCDR1 loop. Non-CDR residues, as defined by each of the numbering schemes, are highlighted in blue. The pivot point residues (L27b in Kabat and L29 in other schemes) are emphasized in bold. Residues for which insufficient sequential statistical information was available are shaded in grey (<30% occupancy). Additionally, highly conserved Cysteine and Tyrosine residues located at the termini of the LCDR1 have been color-coded in yellow and magenta, respectively.

SUPPLIEMENTARY TABLE S8b

Table of Relative Entropy of the Residues in HCDR1 based on different numbering schemes

Kabat		Chothia		AbM		IMGT	
H22	3.938	H22	3.938	H22	3.938	23	3.938
H23	1.241	H23	1.241	H23	1.241	24	1.241
H24	1.544	H24	1.544	H24	1.544	25	1.544
H25	1.776	H25	1.776	H25	1.776	26	1.776
H26	1.835	H26	1.835	H26	1.835	27	1.835
H27	1.518	H27	1.518	H27	1.518	28	1.518
H28	1.192	H28	1.192	H28	1.192	29	1.192
**H29**	**2.192**	**H29**	**2.192**	**H29**	**2.192**	**30**	**2.267**
H30	0.951	H30	0.951	H30	0.951	31	0.896
H31	0.839	H31	0.839	H31	0.839	32	0.776
		H31a	-	H31a	-	32.1	-
		H31b	-	H31b	-	32.2	-
		H31c	-	H31c	-	35	0.896
		H31d	-	H31d	-	36	0.776
H32	1.337	H32	1.597	H32	1.597	37	1.597
H33	0.582	H33	0.706	H33	0.706	38	0.706
H34	1.998	H34	2.298	H34	2.298	39	2.298
H35	1.271	H35	1.443	H35	1.443	40	1.443
H36	3.363	H36	3.363	H36	3.363	41	3.363

Supplemental Table S8b. The relative entropy of conservation calculated in HCDR1 loop. Non-CDR residues, as defined by each of the numbering schemes, are highlighted in blue. The pivot point residues (H30 in IMGT and H29 in other schemes) are emphasized in **bold**. Residues for which insufficient sequential statistical information was available are shaded in grey. Additionally, highly conserved Cysteine and Tyrosine residues located at the termini of the LCDR1 have been color-coded in yellow and magenta, respectively. Note that in Kabat numbering scheme, the insertion site was placed at H35 as no residue was equivalent to the H31a-d residues. Additionally, in the IMGT numbering scheme, the H33 and H34 residues lack equivalent residues in the other numbering schemes. These residues also lack sufficient database information, with both residues having <30% occupancy.

SUPPLIEMENTARY TABLE S9a

Table of the Average Relative Entropy Calculated in each Regions in the Variable Domains between Human and Mouse

Region	Avg. RE	Region	Avg. RE
LFR-1	0.496	HFR-1	0.396
LFR-2	0.395	HFR-2	0.425
LFR-3	0.396	HFR-3	0.517
LFR-4	0.456	HFR-4	0.219
LCDR1	0.754	HCDR1	0.437
LCDR2	0.676	HCDR2	0.561
LCDR3	0.884	HCDR3	0.315
L-Frameworks	0.432	H-Frameworks	0.422
LCDRs	0.776	HCDRs	0.450
Light Chain	0.520	Heavy Chain	0.431

Supplemental Table S 9a. The average relative entropy calculated in different regions (Kabat Numbering scheme) for the Homo sapiens immunoglobulin variable domains, with the mouse variable domains as the reference states.

SUPPLIEMENTARY TABLE S9b

Table of the Average, Median, Standard Deviation of the Relative Entropy Calculated and outliers in each Regions in the Variable Domains between Human and Mouse

**Region**	**Avg. RE** (Differences)	**Mdn. RE** (Differences)	**SD. RE** (Differences)	**Outliers** (RE)
LFR-1	0.496	0.324	0.526	L2 (2.026)
				L11 (1.397)
LFR-2	0.395	0.166	0.654	L43 (2.658)
LFR-3	0.396	0.165	0.506	L66 (2.261)
				L81 (1.341)
LFR-4	0.456	0.193	0.595	L100 (1.832)
HFR-1	0.396	0.223	0.369	H5 (1.104)
				H19 (1.184)
HFR-2	0.425	0.176	0.561	H40 (1.853)
HFR-3	0.506	0.430	0.517	H67 (1.815)
				H75 (1.632)
				H84 (1.545)
HFR-4	0.219	0.074	0.304	H108 (0.952)
LCDR1	0.754	0.438	0.935	L27B (3.394)
LCDR2	0.676	0.327	0.663	L51 (1.511)
				L55 (1.757)
LCDR3	0.884	0.768	0.553	L90 (2.066)
HCDR1	0.437	0.259	0.403	H27 (1.348)
HCDR2	0.561	0.515	0.345	H52A (1.423)
				H60 (1.116)
HCDR3	0.315	0.264	0.278	H102 (1.166)

Supplemental Table S9b. The average (Avg), median (Mdn) and standard deviation (SD) of the relative entropies of differences between mouse and human calculated in different regions (Kabat Numbering scheme) in the Homo sapiens immunoglobulin variable domains. The residues with RE value higher than the average RE plus 1.65 times the standard deviation were identified as the outliers in the group.

SUPPLIEMENTARY TABLE S10

Table of the Amino Acid Distributions at the Outlier Residues in Human and Mouse

Region	Residue	RE (Diff.)	Homo sapiens AA Distribution	Mus Musculus AA Distribution
LFR-1	L2	2.026	I (42%), S (34%), Y (8%)	I (71%), V (15%) A (6%)
	L11	1.397	L (53%), V (37%), A (7%)	L (63%), M (31%)
LCDR1	L27B	3.394	N (45%), D(19%), L (14%), V (9%),	L (50%), V (20%). I (14%), A (13%)
LFR-2	L43	2.658	A (76%), S (10%), P (8%)	S (65%), P (15%), T (9%)
LCDR2	L51	1.511	A (42%), N (20%), D (11%), V (11%), T (6%)	T (39%), A (37%), V (16%)
	L55	1.757	P (42%), A (23%), Q (15%), E (10%),	A (35%), E (18%), F (13%), H (7%), Y (6%), D (6%)
LFR-3	L66	2.261	G (51%), K (30%)	G (89%), L (6%)
	L83	1.341	E (45%), F (37%), V (12%)	L (33%), A (26%), V (9%), I (8%), E (6%)
LCDR3	L90	2.066	Q (47%), S (17%), T (14%), A (8%),	Q (79%), L (7%), H (6%), N (6%),
LFR-4	L100	1.832	G (36%), Q (33%), T (17%)	G (62%), A (22%), S (13%)
HFR-1	H5	1.104	V (52%), L (19%), Q (17%), E (5%),	Q (79%),V (10%)
	H19	1.184	R (52%), K (22%), S (20%)	K (88%), S (13%)
HCDR1	H27	1.348	F (48%), Y (18%), G (17%), D (7%),	Y (66%), F (30%)
HFR-2	H40	1.853	A (65%), P (17%), S (6%)	R (56%), S (13%), T (9%), P (9%), A (6%)
HCDR2	H52A	1.423	P (27%), G (17%) Y (15%), S (13%), A (6%),	P (79%), S (9%)
	H60	1.116	A (60%), N (16%), S (13%)	N (72%) A (9%), P (6%)
HFR-3	H67	1.815	F (47%), V (40%), L (6%), I (5%),	A (66%), F (18%), L (8%)
	H75	1.632	K (62%), T (12%), I (9%)	S (68%), K (23%)
	H84	1.545	A (53%), S (19%), P (10%), V (5%)	S (77%), T (12%), A (6%)
HCDR3	H102	1.166	Y (33%), V (24%), I (9%), P (8%), L (8%)	Y (79%), V (14%)
HFR-4	H108	0.952	L (63%), T (22%), M (9%)	T (53%), S (24%), L (21%)

Supplemental Table S10. The amino acid frequencies (>5%) of the outliers based on the relative entropy differences between the mouse and the human immunoglobulin variable domain.

SUPPLIEMENTARY TABLE S11

Table of the Residue Relative Entropy, Consensus Mouse variable domain Sequences and Calculated AbNatiV Score based on the consensus sequence

VL	Hc	Mc	RE	(1-AbN Score) kappa	(1-AbN Score) lambda	VH	Hc	Mc	RE	(1-AbN Score)
L1	**D**	**D**	0.518	0.000	**0.076**	H1	**Q**	**Q**	0.070	0.000
L2	**I**	**I**	**2.026**	0.000	**0.076**	H2	**V**	**V**	0.120	0.000
L3	**V**	**V**	0.471	**0.072**	0.000	H3	**Q**	**Q**	0.185	0.000
L4	L	M	0.195	0.000	**0.088**	H4	**L**	**L**	0.018	0.000
L5	**T**	**T**	0.055	0.000	0.000	H5	V	Q	**1.104**	**0.091**
L6	**Q**	**Q**	0.045	0.000	0.000	H6	E	Q	0.223	0.000
L7	**S**	**S**	1.221	0.000	**0.040**	H7	**S**	**S**	0.659	0.000
L8	**P**	**P**	0.167	0.000	**0.091**	H8	**G**	**G**	0.012	0.000
L9	S	A	0.934	**0.084**	**0.073**	H9	G	A	0.452	0.000
L10	**S**	**S**	0.593	0.000	0.001	H10	G	E	0.496	0.000
L11	**L**	**L**	**1.397**	0.000	0.000	H11	**L**	**L**	0.998	**0.091**
L12	**S**	**S**	0.322	0.000	0.000	H12	**V**	**V**	0.689	**0.089**
L13	A	V	1.254	0.003	0.000	H13	**K**	**K**	0.369	0.000
L14	**S**	**S**	0.430	**0.041**	**0.032**	H14	**P**	**P**	0.008	0.000
L15	P	L	0.383	**0.064**	**0.069**	H15	**G**	**G**	0.094	0.000
L16	**G**	**G**	0.010	0.000	0.000	H16	G	A	0.915	0.000
L17	Q	E	0.299	**0.058**	**0.055**	H17	**S**	**S**	0.188	0.001
L18	**R**	**R**	0.332	0.000	**0.064**	H18	L	V	0.598	0.000
L19	**V**	**V**	0.074	0.000	0.005	H19	**K**	**K**	**1.184**	0.000
L20	**T**	**T**	0.293	0.000	0.000	H20	**L**	**L**	0.462	**0.021**
L21	**I**	**I**	0.324	0.000	0.000	H21	**S**	**S**	0.044	0.000
L22	S	T	0.065	0.000	0.000	H22	**C**	**C**	0.002	0.000
L23	**C**	**C**	0.007	0.000	0.000	H23	A	K	0.683	0.003
L24	**R**	**R**	0.287	0.000	**0.059**	H24	**A**	**A**	0.139	0.000
L25	**A**	**A**	1.941	0.000	**0.082**	H25	**S**	**S**	0.181	0.000
L26	**S**	**S**	0.455	0.000	**0.019**	H26	**G**	**G**	0.108	0.000
L27	**Q**	**Q**	0.288	0.002	**0.089**	H27	F	Y	**1.348**	0.000
L27A	**S**	**S**	0.053	**0.015**	0.001	H28	**T**	**T**	0.133	0.000
L27B	N	L	**3.394**	0.000	0.001	H29	**F**	**F**	0.262	0.000
L28	S	N	1.138	0.001	0.055	H30	S	T	0.860	0.001
L29	**G**	**G**	0.438	**0.088**	**0.046**	H31	**S**	**S**	0.125	0.000
L30	**S**	**S**	0.270	**0.090**	**0.090**	H32	**Y**	**Y**	0.180	0.000
L31	N	S	0.390	0.000	0.000	H33	**W**	**W**	0.592	**0.058**
L32	**Y**	**Y**	0.159	0.000	0.000	H34	**M**	**M**	0.255	0.000
L33	**L**	**L**	0.490	0.000	**0.052**	H35	**H**	**H**	0.506	0.000
L34	A	H	0.507	0.002	**0.043**	H36	**W**	**W**	0.002	0.000
L35	**W**	**W**	0.002	0.000	0.000	H37	V	**V**	0.157	0.000
L36	**Y**	**Y**	0.124	0.001	**0.018**	H38	R	K	1.130	**0.088**
L37	**Q**	**Q**	0.123	0.000	0.003	H39	**Q**	**Q**	0.028	0.000
L38	**Q**	**Q**	0.130	0.000	0.000	H40	A	R	**1.853**	**0.085**
L39	**K**	**K**	0.351	0.000	0.000	H41	**P**	**P**	0.128	0.000
L40	**P**	**P**	0.159	0.000	0.000	H42	**G**	**G**	0.313	0.000
L41	**G**	**G**	0.198	0.000	0.000	H43	**K**	**K**	0.314	0.000
L42	**Q**	**Q**	0.360	0.000	0.000	H44	**G**	**G**	0.194	0.005

L43	A	S	2.658	0.012	0.043
L44	P	P	0.166	0.000	0.000
L45	K	K	0.570	0.000	0.086
L46	L	L	0.217	0.000	0.000
L47	L	L	0.700	0.000	0.000
L48	I	I	0.047	0.000	0.000
L49	Y	Y	0.121	0.000	0.000
L50	G	Y	0.436	0.054	0.066
L51	A	T	1.511	0.000	0.004
L52	S	S	0.194	0.000	0.000
L53	N	N	0.327	0.000	0.002
L54	R	L	0.294	0.007	0.088
L55	P	A	1.757	0.084	0.089
L56	S	S	0.214	0.000	0.000
L57	G	G	0.043	0.000	0.000
L58	V	V	0.422	0.000	0.001
L59	P	P	0.086	0.000	0.000
L60	D	D	0.559	0.086	0.000
L61	R	R	0.019	0.000	0.000
L62	F	F	0.010	0.000	0.000
L63	S	S	0.173	0.000	0.000
L64	G	G	0.046	0.000	0.000
L65	S	S	0.016	0.000	0.000
L66	G	G	2.261	0.000	0.042
L67	S	S	0.114	0.000	0.001
L68	G	G	0.072	0.000	0.000
L69	T	T	0.421	0.000	0.000
L70	D	D	1.113	0.000	0.090
L71	F	F	0.812	0.000	0.058
L72	T	T	0.148	0.000	0.002
L73	L	L	0.008	0.000	0.000
L74	T	T	1.079	0.000	0.000
L75	I	I	0.029	0.000	0.000
L76	S	S	0.175	0.000	0.000
L77	G	S	0.636	0.000	0.022
L78	L	V	0.946	0.089	0.009
L79	Q	E	0.448	0.000	0.063
L80	A	A	0.453	0.000	0.000
L81	E	E	0.521	0.000	0.000
L82	D	D	0.005	0.000	0.000
L83	E	L	1.341	0.030	0.091
L84	A	A	0.112	0.081	0.000
L85	D	T	0.451	0.000	0.082
L86	Y	Y	0.001	0.000	0.000
L87	Y	Y	0.157	0.000	0.000
L88	C	C	0.001	0.000	0.000
L89	Q	Q	0.423	0.008	0.025
L90	Q	Q	2.066	0.002	0.055
L91	Y	Y	0.342	0.001	0.061
L92	D	S	0.754	0.000	0.085

H45	L	L	0.018	0.000
H46	E	E	0.057	0.000
H47	W	W	0.013	0.000
H48	I	I	0.637	0.089
H49	G	G	1.110	0.000
H50	R	R	0.575	0.035
H51	I	I	0.196	0.000
H52	S	D	0.705	0.000
H52A	P	P	1.423	0.000
H53	S	N	0.623	0.063
H54	G	S	0.515	0.000
H55	G	G	0.381	0.000
H56	S	G	0.330	0.006
H57	T	T	0.454	0.000
H58	Y	K	0.401	0.006
H59	Y	Y	0.075	0.000
H60	A	N	1.116	0.090
H61	D	E	0.779	0.000
H62	S	K	0.894	0.000
H63	V	F	0.526	0.000
H64	K	K	0.309	0.090
H65	G	G	0.235	0.000
H66	R	K	1.142	0.091
H67	F	A	1.815	0.073
H68	T	T	0.041	0.000
H69	I	L	0.858	0.000
H70	S	T	0.597	0.000
H71	R	V	0.609	0.081
H72	D	D	0.021	0.001
H73	N	K	0.538	0.055
H74	S	S	0.292	0.000
H75	K	S	1.632	0.077
H76	N	S	0.626	0.000
H77	T	T	0.215	0.000
H78	A	A	0.556	0.003
H79	Y	Y	0.827	0.000
H80	L	M	0.412	0.000
H81	Q	Q	0.310	0.090
H82	L	L	0.448	0.000
H82A	N	S	0.272	0.000
H82B	S	S	0.098	0.000
H82C	L	L	0.206	0.000
H83	T	T	0.773	0.000
H84	A	S	1.545	0.000
H85	E	E	0.610	0.000
H86	D	D	0.006	0.000
H87	T	S	0.970	0.090
H88	A	A	0.003	0.000
H89	V	V	0.063	0.000
H90	Y	Y	0.003	0.000

L93	**S**	**S**	0.382	0.000	0.000
L94	S	Y	0.863	0.002	0.001
L95	**P**	**P**	1.387	0.000	0.000
L96	Y	L	0.972	0.000	**0.010**
L97	**T**	**T**	0.768	0.000	**0.090**
L98	**F**	**F**	0.019	0.000	0.000
L99	**G**	**G**	0.003	0.000	0.000
L100	**G**	**G**	**1.832**	0.000	0.000
L101	**G**	**G**	0.007	0.000	0.000
L102	**T**	**T**	0.003	0.000	0.000
L103	**K**	**K**	0.146	0.000	0.000
L104	**L**	**L**	1.218	0.001	0.000
L105	**E**	**E**	0.832	0.000	**0.090**
L106	**I**	**I**	0.793	0.000	**0.091**
L107	**K**	**K**	0.239	0.000	**0.088**
L108	**R**	**R**	0.376	**0.087**	**0.087**

H91	**Y**	**Y**	0.011	0.000
H92	**C**	**C**	0.000	0.000
H93	**A**	**A**	0.024	0.000
H94	**R**	**R**	0.276	0.000
H95	D	Y	0.394	**0.050**
H96	G	Y	0.264	0.003
H97	**Y**	**Y**	0.286	0.002
H98	G	Y	0.257	**0.033**
H99	G	Y	0.124	0.000
H100	G	Y	0.122	**0.060**
H100A	**Y**	**Y**	0.223	0.000
H100B	Y	F	0.330	**0.075**
H100C	Y	Y	0.410	**0.030**
H101	**D**	**D**	0.216	0.000
H102	**Y**	**Y**	**1.166**	0.000
H103	**W**	**W**	0.003	0.000
H104	**G**	**G**	0.002	0.000
H105	**Q**	**Q**	0.419	0.000
H106	**G**	**G**	0.004	0.000
H107	**T**	**T**	0.074	0.000
H108	L	T	**0.952**	**0.033**
H109	**V**	**V**	0.536	0.000
H110	**T**	**T**	0.162	0.000
H111	**V**	**V**	0.016	0.000
H112	**S**	**S**	0.017	0.000
H113	**S**	**S**	0.227	0.000

Supplemental Table S11. The Relative Entropy calculated based on the amino acid distribution differences between Homo sapiens and Mus antibody variable domain sequences. The amino acid consensus Human (Hc) and Mouse (Mc) were listed, with identical sequences in **bold**. The AbNatiV Score (AbN score) of the consensus Mus amino acid sequences represent the humanness score of each residue based on VL kappa, VL lambda and VH models. To align with the relative entropy, we used (1-AbNatiV Score) to depict the nonhumanness level of the sequences. The hotspots identified in the RE analysis and the AbNatiV analysis ((1-AbNative Score) > 0.01) were highlighted in **red**. The CDRs (Kabat definition) were highlighted in red, green and magenta respectively.
